# A Nano‐Electro‐Platform Enabling Evolutionary Screening and Remodeling of Tumor Cells for Metastasis Inhibition

**DOI:** 10.1002/advs.202507684

**Published:** 2025-06-20

**Authors:** Feng Liu, Hong Sun, Bing Liu, Jing Zhang, Chengbao Wu, Shi Yan, Chengzheng Tai, Yihang Tong, Rongtai Su, Xiaowei Xiang, Han Wu, Fuqi Yao, Kuan Yang, Dedong Yin, Yuqiong Wang, Ao Xiao, Long Cheng, Xi Chen, Nan Wu, Zaizai Dong, Lingqian Chang

**Affiliations:** ^1^ School of Engineering Medicine Beihang University Beijing 100191 China; ^2^ Key Laboratory for Biomechanics and Mechanobiology School of Biological Science and Medical Engineering Beihang University Beijing 100191 China; ^3^ Qingdao Research Institute Beihang University Qingdao 266100 China; ^4^ Translational Medicine Center Beijing Chest Hospital Capital Medical University & Beijing Tuberculosis and Thoracic Tumor Research Institute Beijing 101149 China; ^5^ Key Laboratory of Carcinogenesis and Translational Research (Ministry of Education) Department of Thoracic Surgery II Peking University Cancer Hospital & Institute Beijing 100142 China; ^6^ School of Biomedical Engineering Anhui Medical University Hefei 230032 China; ^7^ Department of Ophthalmology Beijing Friendship Hospital, Capital Medical University Beijing 100050 China; ^8^ State Key Laboratory of Molecular Oncology Department of Thoracic Surgery II Peking University Cancer Hospital & Institute Beijing 100142 China

**Keywords:** electroporation, extracellular vesicles, immunotherapy, nano‐chip, tumor metastasis

## Abstract

Tumor metastasis remains the leading cause of mortality among cancer patients. Addressing this challenge necessitates the development of effective strategies for targeted drug delivery and therapy. Given that metastatic lesions are primarily driven by highly aggressive tumor cell subpopulations, in‐depth study of these cells and further guiding design of targeted therapeutics, play deterministic roles in metastasis inhibition. Herein, a nano‐electro‐platform is shown that enables non‐invasive screening of aggressive cell subpopulations from heterogeneous tumor samples. Single‐cell sequencing further reveals immune evasion pathways associated with their aggressive behavior. Targeting the screened aggressive cells, the platform implements a unique nanopore‐focused electric field, which genetically remodels the cells to generate extracellular vesicles (EVs) with significantly enhanced tumor‐targeting and therapeutic capabilities. The engineered EVs effectively activate macrophages and T cells, leading to robust tumor cell elimination and metastasis inhibition in lung cancer metastasis models. These highlight a versatile, multidisciplinary technique adopting a new path toward deep understanding and treating metastasis.

## Graphical abstract

The nano‐electro‐platform achieves non‐destructive evolutionary screening and electro‐remodeling of tumor cells to generate extracellular vesicles with enhanced tumor‐targeting and therapeutic functions. These extracellular vesicles demonstrate remarkable efficacy in activating both macrophages and T cells, thereby orchestrating a potent immune response that results in the elimination of tumor cells and the suppression of metastatic progression within lung cancer metastasis models.

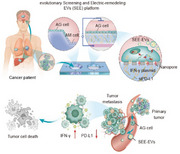

## 1 Introduction

Tumor metastasis is the leading cause of mortality in cancer patients, making mechanism research and effective treatment imperative.^[^ ^1^, ^2^ ^]^ Due to the high heterogeneity of tumors, metastatic foci are predominantly formed by a subpopulation of aggressive cells behaving enhanced migration and invasiveness. Screening the aggressive subgroup for decoding the underlying mechanism brings about important hints for inhibiting tumor metastasis.^[^ ^3^, ^4^, ^5^ ^]^ However, current screening methods including flow cytometry depend on a single or a low number of biomarkers, falling short of precise differentiation of the subgroup orchestrated by a variety of genetic regulation.^[^ ^6^, ^7^ ^]^ Furthermore, based on a limited understanding of the subgroup, traditional therapeutics (i.e., chemotherapy drugs and immunotherapy inhibitors) for inhibiting tumor metastasis face challenges to achieving high efficiency in targeting and precise elimination.^[^ ^8^, ^9^, ^10^, ^11^ ^]^ Technologies that enable precise screening of the highly migratory and invasive subgroups while facilitating the development of targeted drugs have been sought after for the long term yet remained wide open to tumor metastasis inhibition.

Here, we developed a nanoplatform for evolutionary Screening and Electro‐remodeling of tumor cells to generate Extracellular vesicles with enhanced tumor‐targeting and therapeutic functions (“SEE” platform). The platform first adopts a 3D cell‐culture module for mimicking in vivo invasion of cancer cells, to screen aggressive cell subpopulation (AG cells) from the patient's tumor tissue, which bypasses the limit of biomarkers. Single‐cell sequencing next revealed key genes, including carbonic anhydrase IX (*CA9*) and lactamase beta 2 (*LACTB2*) related to immune escape, determining the migratory and invasive variances between AG cells and their non‐aggressive counterparts. Facilitated by the SEE platform, new findings relevant to AG cells further indicate that their released EVs present significantly enhanced capacity to targeting tumors, encouraging a cell‐remodeling strategy for metastasis inhibition by adopting the engineered EVs. To this aim, the SEE platform adopts a nanopore‐focused electric field that not only achieves safe, efficient intracellular delivery of therapeutic molecules for remodeling AG cells but also triggers the remodeled cells to produce therapeutic‐EVs (SEE‐EVs) that efficiently transport into targeted tumor cells. For in vivo application toward metastasis treatment, the SEE platform was applied for remodeling AG cells from lung cancer patients by transfecting immunotherapy promoters, Interferon‐γ (IFN‐γ) plasmid, and Programmed cell death ligand 1(PD‐L1) siRNA. Systematic investigations based on both cell‐derived xenograft (CDX) and patient‐derived xenograft (PDX) models validated the significantly enhanced therapeutic effects of the SEE‐EVs to host immune system, regaining its abilities in identifying and eliminating tumor cells. These highlight a versatile, multidisciplinary nano‐electro‐platform adopting a unique path toward studying and treating tumor metastasis.

## 2 Results

### 2.1 Working Principle of the SEE Platform

**Figure 1:**
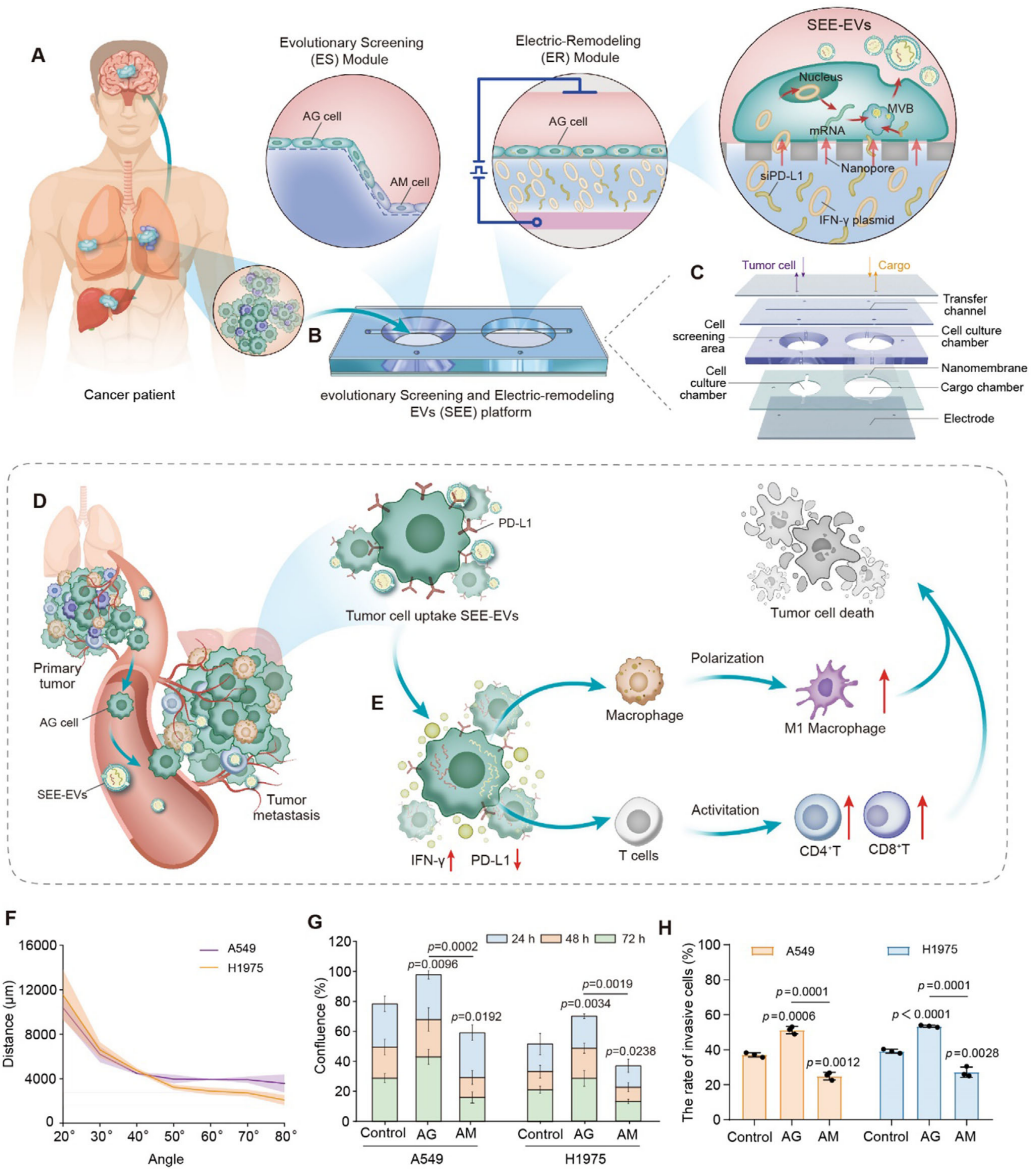
Principle of the SEE platform for metastasis inhibition. A) Acquisition of tumor samples from cancer patient. B) Layout of the SEE platform, including the evolutionary screening (ES) module (left) and electro‐remodeling (ER) module (right). AG cell, aggressive cell. AM cell, ambitionless cell. SEE‐EVs, EVs with tumor‐targeting and therapeutic functions. C) Structural diagram of the SEE platform. D) Schematic diagram illustrating the aggregation of SEE‐EVs inside the tumor. E) Schematic diagram of SEE‐EVs inhibiting tumor metastasis. F) Selection of the slope for evolutionary screening. G) Scratch test to verify the enhanced migration capacity of evolutionarily screened cells. H) 3D invasion assay to verify the increased aggressiveness of the evolutionarily screened cells. The data shown in F‐H were derived from three independent experiments and were presented as mean ± SD; a two‐sided Student's *t*‐test was used for comparisons (F‐H). *p* < 0.05 was considered statistically significant.

The SEE platform consists of an evolutionary screening (ES) module and an electro‐remodeling (ER) module (**Figure** **1**A–C; Figures S1A–F and S2, Supporting Information). The ES module was designed to non‐destructively isolate AG cell subpopulation from patient‐derived tumor tissues. To achieve this, we developed a culture chamber with a flat base and gradient sidewall to simulate the in vivo migration and invasiveness of AG cells from the primary tumor site (flat region) to metastatic niches (gradient region). The gradient region creates a 3D spatial barrier for cell differentiation.^[^ ^12^ ^]^ Due to significant variances on migration and invasiveness, AG cells can be isolated from the gradient region. The subgroup with low migration, named “ambitionless (AM) cells”, remains in the flat region.

Further study on the AG cells isolated by the SEE platform reveals that the EVs released from them have significantly enhanced performance in tumor‐targeting, which encourages adopting the AG‐derived EVs as drug carriers for tumor metastasis inhibition. For developing the engineered therapeutic‐EVs (SEE‐EVs), the ER module is designed on the platform, where a nanopore layer is assembled, forming a unique configuration that generates a focused electric field to safely electroporate the AG cells (cultured on the nanopore layer) while rapidly transporting therapeutic factors into cells. Facilitated by the nanopore‐focused electric field (NEF), the transfected AG cells significantly produce the SEE‐EVs that carry the therapeutic factors.

By using lung cancer as a proof‐of‐concept model, IFN‐γ and PD‐L1 siRNA were applied as therapeutic molecules for restoring the tumor‐killing function of immune cells, including macrophages and T cells, which play crucial roles in eliminating tumor cells. The precursor of IFN‐γ (i.e., IFN‐γ mRNA) was formed by the transcription of IFN‐γ plasmid following its delivery into AG cells and then co‐encapsulated with the delivered PD‐L1 siRNA into the EVs secreted by AG cells. After being reinfused back into the tumor model, the SEE‐EVs efficiently propagate to tumor cells and trigger the intracellular release of IFN‐γ mRNA and PD‐L1 siRNA (Figure 1D). The intracellular IFN‐γ secretion enhanced by IFN‐γ mRNA led to the polarization of macrophages from the M2 phenotype (which promotes tumor growth) to the M1 phenotype (which exerts tumoricidal effects). Meanwhile, the reduced PD‐L1 expression, as inhibited by PD‐L1 siRNA, reactivated the T cells, encompassing both CD4^+^ T cells with the ability to recognize tumor cells and CD8^+^ T cells for subsequent tumor elimination (Figure 1E). Through the dual killing effect (by activating macrophages and T cells), the SEE‐EVs enable effective inhibition of tumor metastasis.

### 2.2 The SEE Platform for Screening Aggressive Cells

To validate the SEE platform for screening AG cells, we focus on lung cancer in this work, in recognition of its high prevalence and metastatic potential.^[^ ^13^, ^14^ ^]^ The screening results of lung cancer cells (A549 and H1975) demonstrate that migratory distance decreases significantly at 30° as compared to 20°, while slopes ≥50°(50°, 60°, 70°, and 80°) show no statistically significant differences in migration distance (Figure 1F; Figure S3A,B, Supporting Information). Accordingly, the slope of 50° was selected for screening AG cells. To further validate the migratory and invasive capacities of AG cells, we compared the cells isolated from 50° to their counterparts remained on the flat region. The results of the scratch assay show a higher confluence of the cells from the slope (Figure 1G; Figure S4A,B, Supporting Information). In addition, the 3D invasion assay suggests an increased rate of invasive cells in the slope group (Figure 1H; Figure S5A–E, Supporting Information), revealing that AG cells exhibit significantly enhanced migration and invasiveness capacities. Additionally, the cells without screening (Control group) exhibit complex features of both the AG cells and AM cells, which implies the heterogeneity of cell behaviors.

To further investigate the mechanisms underlying the enhanced migration and invasiveness capabilities of AG cells, we performed transcriptomic profiling of the AG cells and AM cells. The results highlight 41 upregulated genes and 15 downregulated genes in AG cells of both lung cancer cell lines (Figure SE1A–D, Supporting Information). Among the changed genes, most are closely related to the immune escape of tumors through the inhibition of macrophages and T cells, such as *CA9* ^[^ ^15^, ^16^ ^]^ and *LACTB2*.^[^ ^17^, ^18^ ^]^ These results indicate that the enhanced capabilities of AG cells are mainly derived from the regulation of the immune microenvironment. Transcriptomic profiling further reveals hundreds of personalized genes in determining the aggressive behaviors in the two lung cancer cell lines (Figures SE1E–J and S6A–F, Supporting Information). These data confirm the genetic heterogeneity of cells while indicating the independence of biomarker limitations by adopting the SEE platform for screening AG cells.

Taken together, the SEE platform with a slope‐shaped ES module effectively screens aggressive cell subpopulations with enhanced migration and invasiveness from lung cancer cells. Further investigations identify key genes governing the aggressive behaviors of AG cells, primarily implicated in tumor immune evasion. These findings elucidate potential tumor metastasis mechanisms and offer insights for subsequent drug development strategies.

### 2.3 AG Cell‐Derived EVs with Enhanced Tumor‐Targeting Ability

**Figure 2:**
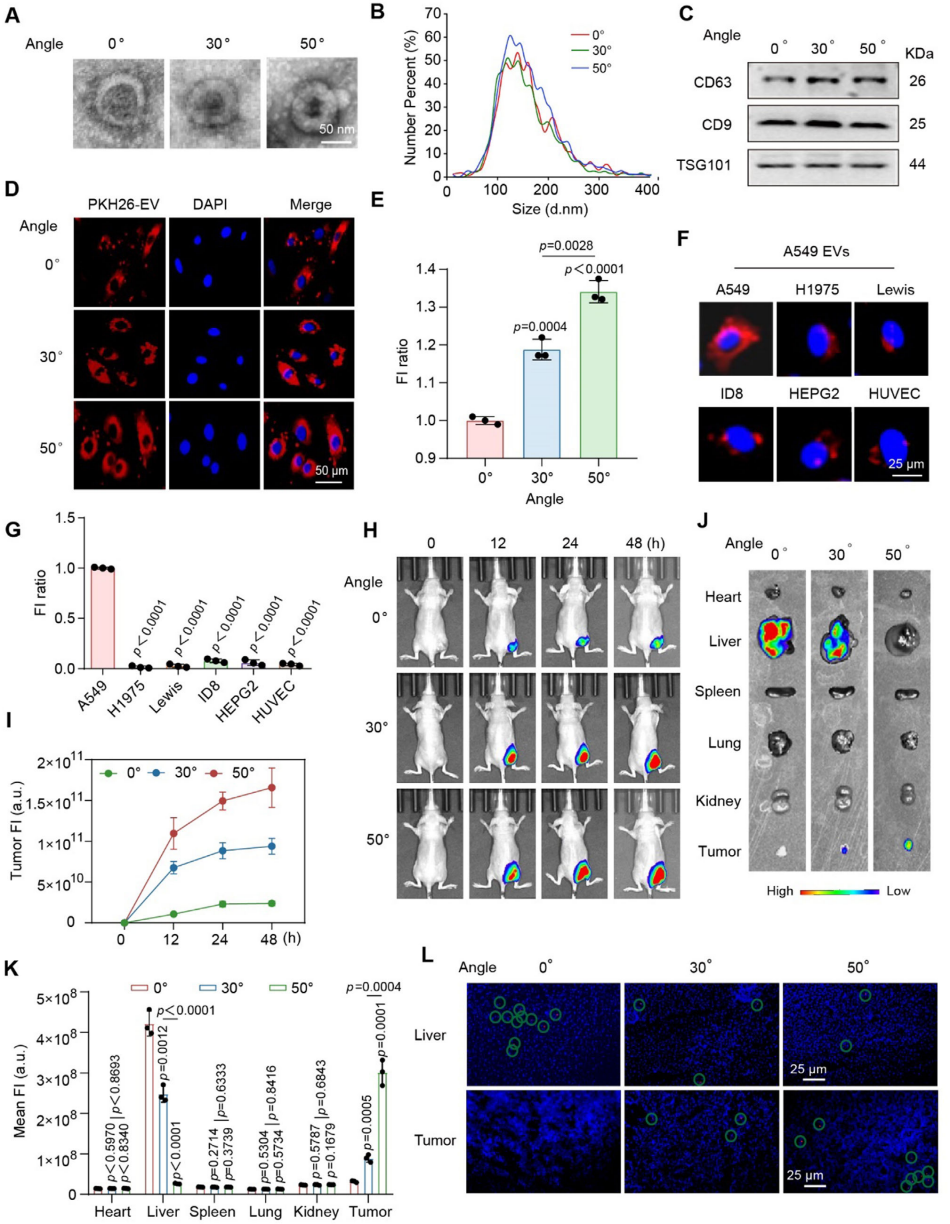
Evolutionary screening enhanced the targeting of EVs to tumor cells. A) TEM images of EVs (scale bars = 50 nm). 0°, 30°and 50° represents EVs derived from cells screened at the slopes of 0° (EVs‐0°), 30° (EVs‐30°) and 50° (EVs‐50°), respectively. B) NTA analysis of EVs. C) Western blot analysis of marker proteins of EVs (CD63, CD9, and TSG101). D,E) Fluorescence images (D) and quantitative analysis (E) of the uptake of EVs by parental A549 cells (scale bars = 50 µm). Red: PKH26‐labeled EVs; Blue: DAPI‐stained cell nuclei. F,G) Fluorescence images (F) and quantitative analysis (G) of the uptake of EVs‐50° by different cell types (scale bars = 25 µm). Red: PKH26‐labeled EVs; Blue: DAPI‐stained cell nuclei. H,I) In vivo distribution (H) and quantitative analysis (I) of EVs in A549 tumor‐bearing BALB/c nude mice at different time points after intravenous injection. J,K) Ex vivo fluorescence images (J) and quantitative analysis (K) of EVs in the main organs from A549 tumor‐bearing BALB/c nude mice. L) Frozen sections of the liver and tumor. The nuclei and EVs are indicted by blue and red fluorescence (marked with green circles), respectively (scale bars = 25 µm). The data shown in E, G, and K were derived from three independent experiments and were presented as mean ± SD; a two‐sided Student's *t*‐test was used for comparisons (E,G,K). *p* < 0.05 was considered statistically significant.

Recent studies have indicated enhanced tumor‐targeting potentials of cancer cell‐derived EVs, along with additional advantages of high tissue penetration and low immunogenicity.^[^ ^19^, ^20^ ^]^ These encouraged us to investigate whether the EVs derived from the SEE‐screened AG cells exhibit functional strengths as a delivery system for metastasis inhibition. For this purpose, EVs from the cells isolated from the SEE platform with 30° (EVs‐30°), 50° slopes (EVs‐50°), and the cells without screening (EVs‐0°) were compared in parallel. The results show no significant differences on morphology (**Figure** **2**A; Figure S7A, Supporting Information), size distribution (Figure 2B; Figure S7B,C, Supporting Information), and expression of EV markers (CD63, CD9, TSG101) (Figure 2C; Figure S7D, Supporting Information). However, the cellular internalization efficiency of EVs from the cells isolated from the SEE platform is significantly increased, in direct proportional to the screening slope gradient (Figure 2D,E; Figure SE2A–D, Supporting Information). Furthermore, parental cells co‐cultured with EVs‐50° resulted in an 8.95‐fold increase in EVs uptake, as compared to that of non‐parental cells (Figure 2F,G; Figure SE2E–H, Supporting Information).

For further validation of the uptake difference in vivo, we established murine tumor models and injected EVs‐0°, EVs‐30°, and EVs‐50° respectively via the tail vein. Notably, EVs‐30° and EVs‐50° exhibit significantly higher accumulation in tumor tissues compared to EVs‐0°, with EVs‐50° demonstrating the most efficient tumor‐targeting capacity. By contrast, EVs‐0° predominantly accumulate in the liver (Figure 2H–L; Figure SE2I–R, Supporting Information). These results demonstrate the enhanced tumor‐targeting of AG cell‐derived EVs.

**Figure 3:**
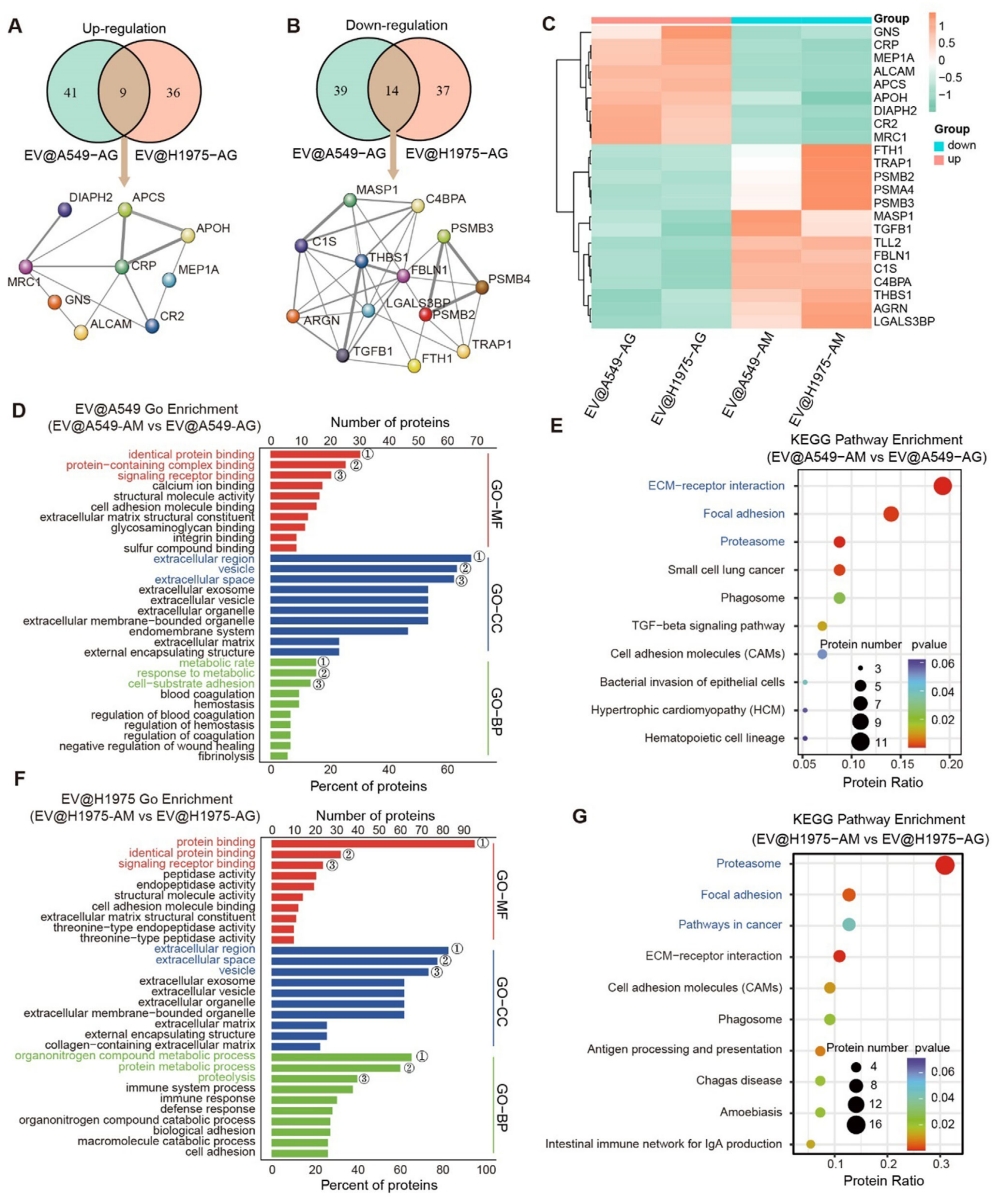
Differential proteomic analysis of EVs derived from evolutionarily screened cells. A,B) Venn diagram of up‐(A) and downregulated (B) proteins in EVs from AG cells (EV@cell‐AG) versus EVs from AM cells (EV@cell‐AM). Coloring based on cluster identifiers. The left circles represent the number of up‐regulated (A) and down‐regulated (B) proteins in EV@A549‐AG versus EV@A549‐AM, respectively. The right circles represent the number of up‐regulated (A) and down‐regulated (B) proteins in EV@H1975‐AG versus EV@H1975‐AM, respectively. Overlapping segments represent the number of genes that are jointly up‐regulated (A) and down‐regulated (B) in the two groups. C) The up‐(orange) and downregulated (green) proteins in EV@cell‐AG versus EV@cell‐AM. D,E) GO (D) and KEGG (E) pathway enrichment analysis of differently expressed proteins between EV@A549‐AM and EV@A549‐AG. F,G) GO (F) and KEGG (G) pathway enrichment analysis of differently expressed proteins between EV@H1975‐AM and EV@H1975‐AG. The screening criterion was fold‐change > 1.5‐fold and *p* < 0.05 were considered statistically significant. *P*‐values were calculated using fisher's exact test with the hypergeometric algorithm and adjusted using the Benjamini‐Hochberg method for multiple tests (D–G).

To investigate the mechanisms underlying the tumor‐targeting of EVs derived from AG cells (EVs‐AG), we performed proteomic profiling on EVs‐AG and EVs derived from AM cells (EVs‐AM). The results show that EVs‐AG in both A549 and H1975 cell lines commonly exhibit 9 upregulated proteins and 14 downregulated proteins (**Figure** **3**A–C). The differentially expressed proteins are mainly involved in protein recognition and cellular binding, with predominant localization in the extracellular region, vesicles, and extracellular space. In terms of biological processes, the identified proteins are mainly linked to metabolic regulation (Figure 3D–G). Additionally, over 40% of the differentially expressed proteins participate in biological activities through secretory mechanisms, suggesting a key role in extracellular signaling and intercellular communication. (Figure S8A,B, Supporting Information).

Taken together, the EVs derived from the SEE‐isolated AG cells exhibit significantly enhanced tumor‐targeting efficiency, showing potentials as a delivery system for targeted metastasis inhibition. The study of proteomic profiling reveals that their enhanced affinity toward tumor cells mainly derives from the regulation of proteins related to cellular recognition and binding.

### 2.4 The SEE‐Mediated AG Cell Electro‐Remodeling for Engineering Therapeutic EVs

**Figure 4:**
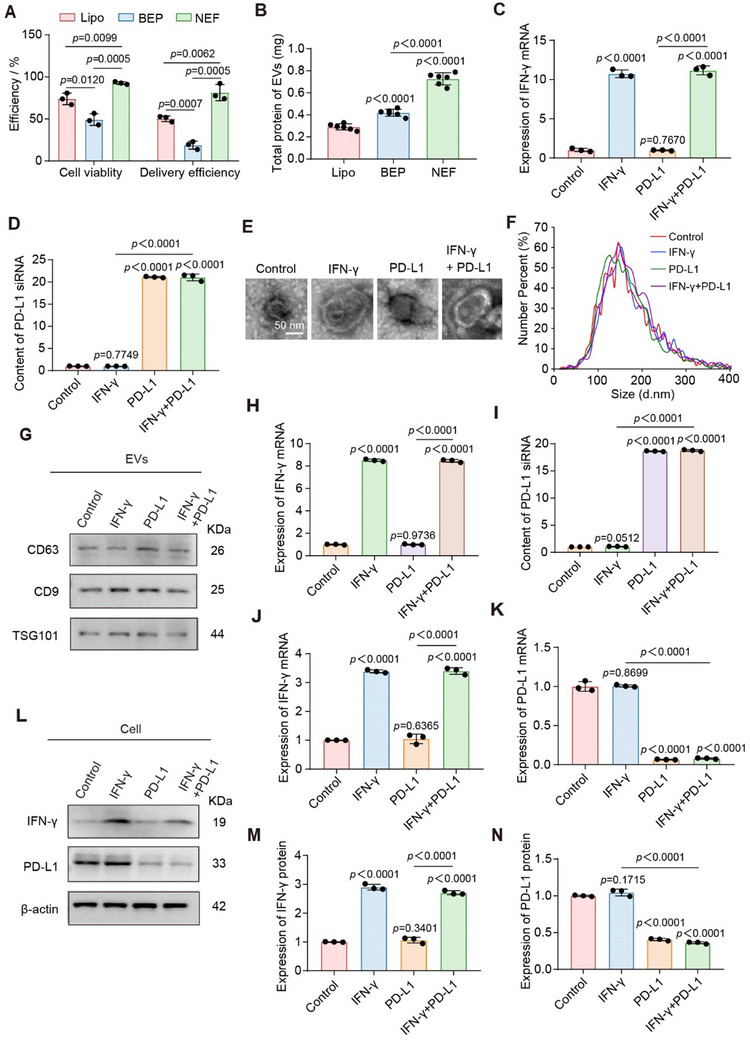
Characterization of EVs endowed with therapeutic properties. A) Cell viability and efficiency of the delivery of GFP plasmid into A549 cells using Lipo, BEP, and NEF, respectively. B) Total protein of EVs from A549 cells after the delivery using Lipo, BEP, and NEF, respectively (*n* = 6). C,D) Content of IFN‐γ mRNA (C) and PD‐L1 siRNA (D) in A549 cells after delivery using NEF. Control: untreated cells; IFN‐γ: cells loaded with the IFN‐γ plasmid; PD‐L1: cells loaded with PD‐L1 siRNA; IFN‐γ+PD‐L1: cells loaded with both the IFN‐γ plasmid and PD‐L1 siRNA. E) TEM images of EVs (scale bars = 50 nm). (E–I) Control: EVs derived from untreated cells; IFN‐γ: EVs derived from cells loaded with the IFN‐γ plasmid; PD‐L1: EVs derived from cells loaded with PD‐L1 siRNA; IFN‐γ+PD‐L1: EVs derived from cells loaded with both the IFN‐γ plasmid and PD‐L1 siRNA. F) NTA analysis of EVs. G) Western blot analysis of marker proteins of EVs (CD63, CD9, and TSG101). H,I) Content of IFN‐γ mRNA (H) and PD‐L1 siRNA (I) in EVs. J,K) Content of IFN‐γ mRNA (J) and PD‐L1 mRNA (K) in parental cells after incubation with EVs. (J–N) Control: parental cells after incubation with EVs derived from untreated cells; IFN‐γ: parental cells after incubation with EVs derived from cells that were loaded with the IFN‐γ plasmid; PD‐L1: parental cells after incubation with EVs derived from cells that were loaded with PD‐L1 siRNA; IFN‐γ+PD‐L1: parental cells after incubation with EVs derived from cells that were loaded with both the IFN‐γ plasmid and PD‐L1 siRNA. L) Western blot analysis of IFN‐γ and PD‐L1 protein levels in parental cells after incubation with EVs. M,N) Quantitative analysis of IFN‐γ (M) and PD‐L1 protein levels (N) in parental cells after incubation with EVs. The data were derived from three independent experiments unless otherwise stated and were presented as mean ± SD; a two‐sided Student's *t*‐test was used for comparisons (A–D, H–K, M and N). *p* < 0.05 was considered statistically significant.

Encouraged by the enhanced tumor‐targeting capability of the AG cell‐derived EVs, we applied the SEE platform for further engineering AG cells to produce therapeutic EVs. To achieve this, the SEE platform adopts the ER module that was designed for transfecting AG cells with therapeutic factors by a nanopore‐focused electric field (NEF). The simulation of the electric field distribution demonstrates that the NEF reaches transmembrane potential at a low voltage, enabling transient and reversible opening of the cell membrane.^[^ ^21^, ^22^, ^23^ ^]^ Meanwhile, charged molecules could be directionally transported into the cell (Figure S9A–D, Supporting Information). Compared to traditional cell‐engineering methods, including liposome (Lipo)^[^ ^24^ ^]^ and bulk electroporation (BEP),^[^ ^25^ ^]^ the SEE platform demonstrates higher cell viability (up to 96%) and delivery efficiency (up to 93%) (**Figure** **4**A; Figure S10A–H, Supporting Information). Furthermore, the SEE platform significantly enhances EVs production, achieving a 2.49‐fold increase compared to Lipo and a 1.60‐fold increase relative to BEP (Figure 4B; Figure S11A–H, Supporting Information). This may be attributed to the NEF‐based delivery on the SEE platform, which maintains high cell viability while upregulating intracellular molecules associated with EV production.^[^ ^26^, ^27^ ^]^

In terms of the therapeutic molecules, given the critical link between immune escape gene networks and AG cell invasiveness,^[^ ^28^, ^29^, ^30^ ^]^ we selected a therapeutic strategy targeting two pathways: macrophages^[^ ^31^, ^32^ ^]^ and T cells.^[^ ^33^, ^34^, ^35^ ^]^ The engineering process comprises three steps, including 1) Intracellular delivery: IFN‐γ plasmid and PD‐L1 siRNA are delivered into screened AG cells to increase intracellular levels of IFN‐γ mRNA and PD‐L1 siRNA; 2) EV secretion: AG cells secret EVs loaded with IFN‐γ mRNA and PD‐L1 siRNA, termed “SEE‐EVs”; 3) Therapeutic delivery: leveraging the high tumor‐targeting capability of SEE‐EVs in vivo, IFN‐γ mRNA and PD‐L1 siRNA are transported into tumor cells, resulting in enhanced IFN‐γ secretion and downregulation of PD‐L1 expression.

We first validated whether these cells were genetically engineered by quantifying RNA levels. The significant increase in intracellular IFN‐γ mRNA (>8.43‐fold) and PD‐L1 siRNA (>19.32‐fold) confirms the implementation of cell engineering (Figure 4C,D; Figure SE3A–D, Supporting Information). Furthermore, in cells receiving exclusive delivery of either IFN‐γ plasmid or PD‐L1 siRNA, the upregulated molecules correspond to the delivered payloads. We then collected EVs derived from the engineered cells and characterized their properties. The results show no significant differences in morphology (Figure 4E; Figure SE3E, Supporting Information), size distribution (Figure 4F; Figure SE3F,G, Supporting Information), or expression of EV marker proteins (CD63, CD9, and TSG101) (Figure 4G; Figure SE3H, Supporting Information) between EVs from engineered cells and unmodified cells. The EVs derived from the cells electro‐remolded with the IFN‐γ plasmid and PD‐L1 siRNA exhibit significantly elevated levels of IFN‐γ mRNA (>6.52‐fold) and PD‐L1 siRNA (>18.03‐fold) (Figure 4H,I; Figure SE3I–L, Supporting Information), demonstrating efficient packaging of therapeutic molecules into SEE‐EVs. To further validate the modulatory effects of SEE‐EVs on tumor cells, we incubated tumor cells with SEE‐EVs and assessed IFN‐γ and PD‐L1 expression. The treated tumor cells exhibited a significant increase of IFN‐γ mRNA (>2.27‐fold) (Figure 4J; Figure SE3M,N, Supporting Information) alongside a marked reduction in PD‐L1 mRNA (>12.42‐fold) (Figure 4K; Figure SE3O,P, Supporting Information). Correspondingly, protein analysis revealed elevated IFN‐γ (>2.71‐fold) and suppressed PD‐L1 expression (>1.43‐fold) (Figure 4L–N; Figure SE3Q–V, Supporting Information).

Taken together, these results demonstrate that the procedure of SEE‐mediated electro‐transfecting IFN‐γ plasmid and PD‐L1 siRNA into AG cells, followed with efficient co‐loading of IFN‐γ mRNA and PD‐L1 siRNA in SEE‐EVs. The engineered SEE‐EVs increase the IFN‐γ and downregulate the PD‐L1 of targeted tumor cells.

### 2.5 The SEE‐EVs Mediated Metastasis Inhibition in CDX Model

To validate the capacity of SEE‐EVs in suppressing tumor metastasis in vivo, we evaluated their therapeutic efficacy in the CDX model. Corresponding to clinical situations, two application scenarios were established by the CDX model, including metastasis prevention and metastasis treatment (see details in Experimental Section). Metastasis prevention refers to pharmacotherapy post‐resection to prevent metastasis originating from incompletely resected tumor remnants or circulating tumor cells, primarily applied to patients deemed eligible for surgical tumor resection. Metastasis treatment refers to direct drug administration for patients diagnosed with pre‐existing metastatic tumors.^[^ ^1^, ^9^ ^]^

**Figure 5:**
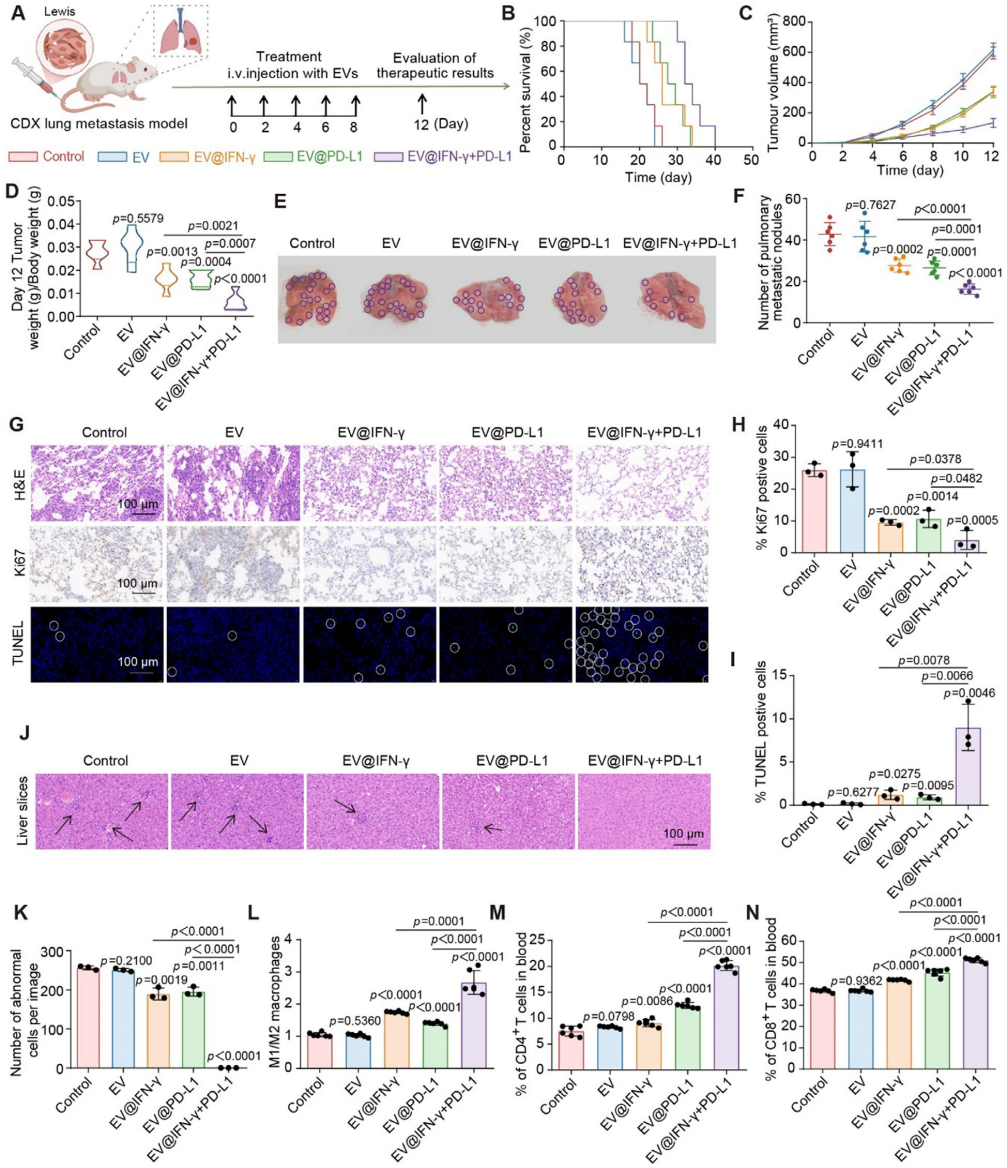
Tumor metastasis inhibition by SEE‐EVs in CDX model. A) The construction and therapeutic regimen of CDX model mice with a risk of tumor metastasis (*n* =  6 mice per group). B) Survival curves of CDX model mice after different treatments. C,D) Growth curves of tumor volume (C) and tumor to body weight ratios (D) of different treatment groups. E) Photographs of lung tissue after different treatments. The purple circles indicate the location of metastatic nodules. F) Number of lung metastatic nodules detected in each group. G) H&E (top), Ki67 (middle), and TUNEL staining (bottom) of lung tissues (scale bars = 100 µm). The white circles indicate TUNEL‐positive cells. H,I) Percentage of Ki67 (H) and TUNEL (I) positive cells among all cells after the treatment ended in each group. J) Images of H&E staining of liver tissue sections after different treatments (scale bars = 100 µm). The black arrows indicate more aggregated abnormal cells. K) Number of abnormal cells in liver tissue after different treatment. L) Phenotypic proportion of peripheral blood macrophages in different treatment groups. M,N) Percentage of CD4^+^ T cells (M) and CD8^+^ T cells (N) in different treatment groups. The data were derived from three independent experiments unless otherwise stated and were presented as mean ± SD; a two‐sided Student's *t*‐test was used for comparisons (D, F, H, I and K–N). *p* < 0.05 was considered statistically significant.

In the CDX metastasis prevention scenario, mice were divided into five groups (n = 6) according to the therapeutic agents: Control (PBS), EV (EVs derived from untreated AG cells), EV@IFN‐γ (EVs derived from AG cells loaded with IFN‐γ mRNA), EV@PD‐L1 (EVs derived from AG cells loaded with PD‐L1 siRNA), and EV@IFN‐γ+PD‐L1 (EVs derived from AG cells loaded with both the IFN‐γ mRNA and PD‐L1 siRNA) (**Figure** **5**A). The results show that the EVs co‐modified with IFN‐γ mRNA and PD‐L1 siRNA significantly improve survival and tumor suppression. The single‐factor engineered EVs exhibit moderate therapeutic effects, and the EV group shows no differences compared to the control group (Figure 5B–D; Figure S12A–G, Supporting Information).

To further evaluate EV‐induced inhibition of tumor metastasis, pulmonary metastatic burden was analyzed. According to lung nodules, the EV@IFN‐γ+PD‐L1 group leads to the lowest number among all groups (Figure 5E,F). In addition, the lowest cell proliferation rate and the highest apoptosis rate are demonstrated in this group by the histopathological assays such as H&E staining, Ki67 (proliferation marker), and TUNEL (cell apoptosis) (Figure 5G–I). Assessment of extrapulmonary metastasis reveals that the EV@IFN‐γ+PD‐L1 group displays no visible metastatic lesions through H&E staining, while the EV@IFN‐γ and EV@PD‐L1 groups show a small number of mitigated lesions in the liver. By contrast, the control/EV groups exhibit prominent hepatic metastases, accompanied by abnormal hepatic function indicators (AST and ALB) (Figure 5J,K; Figure S13A–I, Supporting Information). To investigate the reasons for the variations in treatment outcomes, we analyzed the therapeutic pathways, including macrophage polarization and T cell activation in peripheral blood. The results indicate that the EV@IFN‐γ+PD‐L1 group exhibits the most pronounced shift toward M1 macrophages and the highest frequency of activated T cells (Figure 5L–N; Figure S14A–C, Supporting Information).

For the metastasis treatment scenario, mice were randomly divided into five groups receiving the same treatment regimens as in the metastasis prevention scenario (Figure SE4A, Supporting Information). Following treatment, the engineered EVs (EV@IFN‐γ, EV@PD‐L1, and EV@IFN‐γ+PD‐L1) lead to significantly smaller volumes of tumors as compared to the control and EV groups. EV@IFN‐γ+PD‐L1 group results in the most pronounced reduction (Figure SE4B–J, Supporting Information). The histopathological analysis further reveals the lowest proliferation rate and the most apoptotic cells in the EV@IFN‐γ+PD‐L1 group (Figure SE4K–M, Supporting Information). Further, we investigated changes of macrophage subtypes and T cell activation within tumor tissues. Among all groups, the EV@IFN‐γ+PD‐L1 group demonstrates the highest M1 macrophage polarization and the most T cell activation, indicating immune activation driven by the dual therapeutic molecules encapsulated in EV@IFN‐γ+PD‐L1 (Figures SE4N–P and S15A–C, Supporting Information).

Furthermore, we evaluated the biosafety of SEE‐EVs for tumor therapy. H&E staining of major organs (heart, liver, spleen, lungs, kidneys) reveals no histopathological abnormalities or inflammatory infiltration in mice treated with SEE‐EVs (Figure S16A, Supporting Information). Serum biochemical analysis further confirms the absence of hepatotoxicity or nephrotoxicity, with all liver (e.g., AST) and kidney function markers (e.g., BUN) within normal ranges (Figure S16B–I, Supporting Information).

Taken together, the SEE‐EVs demonstrate biocompatibility and safety profile in vivo, suggesting broad applicability for drug delivery. Based on the findings that AG cells orchestrate their immune escape by coordinately inhibiting macrophage and T cell activities, the SEE‐EVs engineered with IFN‐γ mRNA and PD‐L1 siRNA exhibit enhanced therapeutic performance in metastasis prevention and cancer treatment.

### 2.6 The SEE‐EVs Mediated Metastasis Inhibition in PDX Model

Based on the validation in the CDX model, we further conducted in vivo application of the SEE‐EVs for metastasis inhibition in the PDX model, a clinically relevant model that recapitulates tumor heterogeneity and stromal microenvironment of patients.^[^ ^36^, ^37^, ^38^, ^39^ ^]^ Histomorphology profile and serum biochemical analysis first confirmed the in vivo biosafety of the SEE‐EVs (Figure S17A–I, Supporting Information). To further validate the efficacy of the SEE‐EVs in metastasis inhibition, both application scenarios of metastasis prevention and treatment were established.

**Figure 6:**
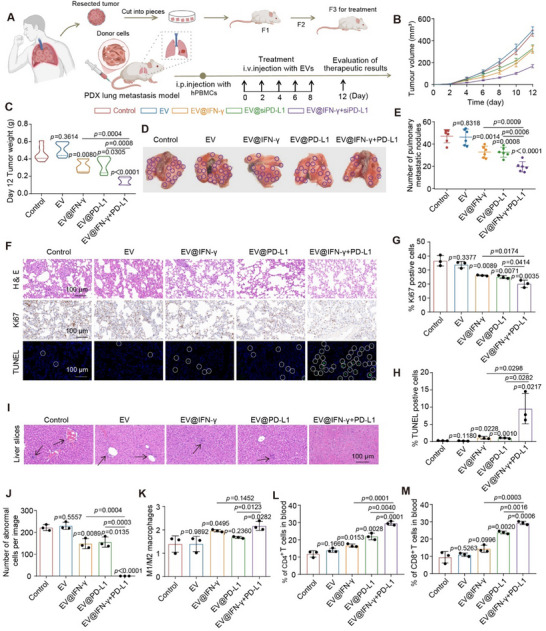
Inhibition of tumor metastasis by SEE‐EVs in PDX model. A) Construction and therapeutic regimen of PDX model mice with a risk of tumor metastasis (*n* =  6 mice per group). B,C) Growth curves of tumor volume (B) and the final weight of tumors (C) in different treatment groups. D) Photographs of lung tissue after different treatment. The purple circles indicate the location of metastatic nodules. E) Number of lung metastatic nodules detected in each group. F) H&E (top), Ki67 (middle), and TUNEL staining (bottom) of lung tissues (scale bars = 100 µm). The white circles indicate the location of TUNEL‐positive cells. G,H) Percentage of Ki67 (G) and TUNEL (H) positive cells among all cells at the end of treatment in each group. I) H&E staining of liver tissues after different treatments (scale bars = 100 µm). The black arrows indicate large aggregations of abnormal cells. J) Number of abnormal cells in liver tissues after different treatments. K) Phenotypic polarization of peripheral blood macrophages in different treatment groups. L,M) Percentage of CD4^+^ T cells (L) and CD8^+^ T cells (M) in different treatment groups. The data were derived from three independent experiments unless otherwise stated and were presented as mean ± SD; a two‐sided Student's *t*‐test was used for comparisons (C, E, G, H, and J–M). *p* < 0.05 was considered statistically significant.

For the metastasis prevention scenario, a portion of tumor tissue was cultured in vitro, followed by AG cell remodeling and SEE‐EVs engineering. The remaining tissue was used to construct the PDX model (**Figure** **6**A; Figure S18A–D, Supporting Information). Upon completion of the model, SEE‐EVs were administered to the mice according to their respective groups for therapeutic efficacy evaluation. Compared to the other four groups, the EV@IFN‐γ+PD‐L1 group demonstrates the highest rate of suppression to tumor growth and the lowest number of metastatic pulmonary nodules (Figure 6B–E; Figure S19A–G, Supporting Information). Additionally, histopathological assays indicate that the EV@IFN‐γ+PD‐L1 group displays optimal pathological outcomes, with the highest efficacy in inhibiting tumor cell proliferation and promoting apoptosis (Figure 6F–H). Assessment of extrapulmonary metastasis reveals no detectable metastases in the EV@IFN‐γ+PD‐L1 group, a low number of lesions in the EV@IFN‐γ and EV@PD‐L1 groups, and a significantly higher number of metastatic lesions in the control and EV groups (Figure 6I–J; Figure S20A, Supporting Information). Serum biochemical analysis also reveals abnormal markers (AST, ALT, and ALB) exceeding the normal threshold in the control and EV groups, whereas the other three treatment groups maintain normal hepatic profiles (Figure S20B–I, Supporting Information). These results validated the therapeutic efficacy of EV@IFN‐γ+PD‐L1 in preventing metastasis. Further analysis demonstrates the synergistic promotion of M1 macrophage polarization and T cell activation in EV@IFN‐γ+PD‐L1 group, which improves its efficacy in tumor elimination (Figure 6K–M; Figure S21A–C, Supporting Information).

Subsequently, we constructed the metastasis treatment scenario for evaluating therapeutic efficacy (Figures SE5A and S22A–D, Supporting Information). The EV@IFN‐γ+PD‐L1 group demonstrates significant suppression of tumor growth among all groups (Figure SE5B–J, Supporting Information). Histopathological evaluation reveals no notable differences between the EV and control groups. The EV@IFN‐γ+PD‐L1 group results in the lowest proliferation rate and highest apoptotic activity in tumor cells (Figure SE5K–M, Supporting Information). Further cell analysis reveals the synergetic activation of the immune system through M1 macrophage polarization and T cell‐mediated cytotoxicity, which achieves significantly improved metastasis treatment (Figures SE5N–P and S23A–C, Supporting Information).

Taken together, the application of SEE‐EVs in PDX models demonstrates its adaptability in clinical mimetic systems. Furthermore, these results demonstrate the advantages of synergetic activation of macrophage and T cells in tumor metastasis inhibition.

## 3 Discussion

In this work, we developed a nano‐electro‐platform addressing the challenge in cancer therapy‐tumor metastasis inhibition. The platform employs an evolutionary screening module for non‐destructive screening the aggressive cell subpopulation. The platform further implements a unique nanopore‐focused electric field, achieving efficient delivery of therapeutic molecules into the AG cells, while triggering the remolded cells to release EVs with enhanced tumor‐targeting and therapeutic properties.

The SEE platform offers distinct advantages over existing methods. 1) For isolating aggressive cell subpopulation, current methods, such as flow cytometry^[^ ^6^ ^]^ and optical tweezers,^[^ ^7^ ^]^ face challenges due to the lack of multiple identification channels for precise cell profiling and screening. The SEE platform, incorporating gradient culture zones, enables cell screening in terms of cellular invasiveness, which bypasses the reliance on biomarkers in conventional approaches. The successful isolation on the SEE platform and molecular characterization of highly invasive cell subpopulations yield mechanistic insights into tumor metastasis, while offering a foundation for developing targeted therapeutic strategies. Considering the wide applicability of the SEE platform for aggressive cell screening, other cells can be expanded for further studies, such as hepatocellular carcinoma cells, a high‐incidence cancer type. 2) Compared with other exogenous carriers‐based drug delivery systems, EVs derived from autologous cells (patient‐derived) minimize immune rejection risks, ensuring biological compatibility.^[^ ^40^, ^41^, ^42^ ^]^ Although tumor cell‐derived EVs have been reported to exhibit targeting capabilities toward tumor in vivo,^[^ ^19^, ^43^ ^]^ in this work, we considered the presence of cellular heterogeneity within tumors while adding the note that the significantly enhanced tumor‐targeting capability EVs derived from the aggressive cell subpopulation. In addition, the inherent diversity of EVs suggests the potential to directly isolate functionally distinct EV subsets from heterogeneous populations for tailored therapeutic applications.^[^ ^44^, ^45^ ^]^ 3) Compared to traditional cell engineering methods,^[^ ^46^ ^]^ the platform‐mediated electro‐remodeling of cells for therapeutic EVs engineering, as evidenced by EVs co‐loaded with IFN‐γ mRNA and PD‐L1 siRNA, presents a novel strategy that overcomes the limitations of conventional non‐targeted drug‐based therapies in suppressing metastatic progression. Given the versatility of the SEE platform for molecular delivery, future improvements could focus on expanding the range of deliverable molecules to enhance therapeutic efficacy through multiple mechanisms.

## 4 Conclusion

Tumor metastasis is the leading cause of mortality in cancer patients, making mechanism research and effective treatment imperative. Due to the high heterogeneity of tumors, metastatic foci are predominantly formed by a subpopulation of aggressive cells behaving enhanced migration and invasiveness. Screening the aggressive subgroup for decoding the underlying mechanism brings about important hint for inhibiting tumor metastasis.

In this work, we developed a nano‐electro‐platform addressing the challenge in cancer therapy‐tumor metastasis inhibition. The platform employs an evolutionary screening module for non‐destructive screening the aggressive cell subpopulation. The platform further implements a unique nanopore‐focused electric field, achieving efficient delivery of therapeutic molecules into the AG cells, while triggering the remolded cells to release EVs with enhanced tumor‐targeting and therapeutic properties that efficiently transport into targeted tumor cells. The engineered EVs effectively activate macrophages and T cells, leading to robust tumor cell elimination and metastasis inhibition in lung cancer metastasis models. These highlight a versatile, multidisciplinary technique adopting a new path toward deep understanding and treating metastasis and overcome the limitations of conventional non‐targeted drug‐based therapies in suppressing metastatic progression.

## 5 Experimental Section

### Fabrication of SEE Platform

The dimensions and structures of each layer of the SEE platform are illustrated in Figure S1 (Supporting Information). Layer (1) was fabricated using polydimethylsiloxane (PDMS, Dow Corning, SylgardTM 184, MI, USA), which was prepared by mixing the elastomer base with curing agent at a 10:1 (w/w) ratio and degassing under vacuum (‐0.1 MPa) for 30 min. After cured at 80 °C for 30 min, the PDMS layer was peeled from the mold and perforated to form inlets and outlets. Layers (2), (3), and (4) were crafted from acrylic plate utilizing laser cutting technology. Layer (5) consisted of indium tin oxide (ITO) glass with a resistance of 6 Ω (NOZO, China). Prior to assembly, layers (1) and (2) were treated with oxygen plasma and bonded at 80 °C for 2 h. Layers (2), (3), (4), (5), and (6) were then assembled using acrylic adhesive. Finally, the prepared chips were rinsed with 75% ethanol and sterile water, followed by UV sterilization for 1 h.

### Cell Culture

Human non‐small cell lung cancer A549 cells were cultured in F‐12K medium (21127022, Gibco, USA) containing 10% fetal bovine serum (FBS) (A5670701, Gibco, USA) and 1% penicillin‐streptomycin (G4015, Servicebio, China). Human non‐small cell lung cancer H1975 cells, human umbilical vein endothelial HUVEC cells, and mouse lung carcinomas Lewis cells were cultured in RPMI 1640 medium (11875119, Gibco, USA) containing 10% FBS and 1% penicillin‐streptomycin. ID8 epithelioid cells and human hepatocellular carcinomas HepG2 cells were cultured in DMEM medium (11965092, Gibco, USA) containing 10% FBS and 1% penicillin‐streptomycin. All cell lines were maintained at 37 °C with 5% CO_2_ in a cell incubator (ThermoFisher Scientific, USA).

### Workflow of SEE Platform

A suspension of 2 × 10^6^ tumor cells was introduced into the ES module via the cell inlet. Following one week of incubation, AG cells migrated upward along the sloped surface. Cell culture medium was then removed and the chip was turned over. 0.05% trypsin solution (G4011, Servicebio, China) was introduced through the cell inlet (not in contact with the cells at the bottom) and incubated with the cells on the slope for 5 min at room temperature. Upon complete digestion of the AG cells, an EVs‐free culture medium was added via the buffer inlet to terminate digestion and facilitate cell transfer into the ER module. The chip was then reverted to its original orientation, enabling cell attachment to the nanomembrane. After a 6 h period, the molecules to be delivered (e.g., 5 µg IFN‐γ plasmid, 20 nm PD‐L1 siRNA (Table S1, Supporting Information) were added through the cargo inlet. A gold needle was pierced into the first layer of the chip to serve as the top electrode and connected to the positive electrode of an electroporator (ECM830, BTX, USA), while the ITO bottom electrode was linked to the negative terminal. Following pulsed electric field application, the top electrode was removed. The cells were subsequently cultured for an additional 48 h before supernatant collection for EVs extraction.

### Cell Scratch Assay

≈2 × 10^6^ cells were uniformly seeded into culture dishes. Upon reaching 80–90% confluency, a straight scratch was introduced across the cell monolayer using a sterile 200 µL pipette tip under a microscope (CKX3‐SLP, OLYMPUS, Japan). Following PBS (G4202, Servicebio, China) washes, the scratch location was marked on the dish bottom for consistent tracking. Serum‐free medium was then added, and the dish was returned to the incubator. Cell migration behavior was monitored and documented at 0 (baseline), 24, 48, and 72 h post‐scratching using phase‐contrast microscopy.

### Cell Invasion Assay

The 3D collagen matrix was prepared using Mouse Tail Collagen Type I Kit (200110‐10, Shengyou, China) following the manufacturer's protocol. Briefly, 200 µL mouse tail collagen type I was mixed with 12 µL 0.1 mol L^−1^ NaOH and 23 µL 10 × PBS (G4202, Servicebio, China). Then, the mixture (100 µL) was evenly spread onto a confocal dish and solidified at 37 °C for 20 min. Next, cells were stained with DAPI (GDP1024, Servicebio, China) for 15 min and seeded onto the collagen matrix for 30 min. The remaining collagen mixture was then overlaid onto the cells. At designated time points, the cells were monitored using a live‐cell imaging station on a fluorescence confocal microscope (Dragonfly, Andor, UK). The rate of invasive cells was calculated as the percentage of invasive cells relative to the total cells.^[^ ^47^, ^48^ ^]^ Invasive cell number was quantified as cells located ± 200 µm vertically from the inoculation plane (Z = 0 µm) (Figure S5A, Supporting Information). The total cell number was the sum of invasive cells and those remaining on the original inoculation plane.

### Collection and Purification of EVs

The cell supernatant containing EVs was sequentially centrifuged at 300 × g for 10 min to remove cells, followed by 2000 × g for 30 min to eliminate cellular debris. The clarified supernatant was then centrifuged at 10 000 × g for 30 min at 4 °C to remove micro‐vesicles. Finally, EVs were isolated via ultracentrifugation at 110 000 × g for 90 min at 4 °C. The morphology of EVs were examined using a transmission electron microscope (JEM‐1400, JEOL, Japan), while their size distribution and concentration were determined by nanoparticle tracking analysis (Zetaview‐PMX120‐Z, Particle Metrix, Meerbusch, Germany).

### Western Blot Analysis

Following the samples lysis with RIPA buffer (PC101, Epizyme Biotech, China), protein concentrations were quantified using a BCA protein assay kit (ZJ102, Epizyme Biotech, China). Then, the protein samples were separated by 12.5% PAGE gel (PG113, Epizyme Biotech, China) and subsequently transferred onto polyvinylidene fluoride (PVDF) membranes (88518, ThermoFisher, USA). The membranes were blocked with 5% BSA (PS113, Epizyme Biotech, China) in Tris‐buffered saline (TBS, PS103, Epizyme Biotech, China) containing 0.1% tween 20 (9005‐64‐5, Beyotime, China) and then incubated overnight at 4 °C with primary antibodies (details are provided in Table S2, Supporting Information). After washing, the membranes were probed with HRP‐conjugated secondary antibodies for 40 min at room temperature. Protein bands were visualized using an enhanced chemiluminescence detection system (GE Healthcare, UK).

### RNA Extraction and RT‐qPCR Assay

Total RNA was extracted using a commercial kit (RC112, Vazyme, China) following the manufacturer's instruction. Target gene expression was analyzed by quantitative reverse transcription PCR (RT‐qPCR) using HiScript II One Step qRT‐PCR SYBR Green Kit (Q221, Vazyme, China). The sequence of all primers is provided in Table S1 (Supporting Information).

### EV Internalization Analysis

EVs were labeled with PKH26 dye (MINI26‐1KT, Sigma, USA) for 10 min at room temperature. The staining reaction was quenched by adding FBS, followed by ultracentrifugation to isolate the PKH26‐labeled EVs. For the uptake assay, 10 µg PKH26‐labeled EVs were co‐incubated with different cells for 12 h. After incubation, unbound EVs were removed by washing, and the cells were fixed with 4% paraformaldehyde (G1101, Servicebio, China). Nuclei were counterstained with DAPI prior to fluorescence imaging.

### Liposome‐Mediated Delivery Assay

The lipofectamine (L3000015, ThermoFisher, USA) was used to deliver plasmid (5 µg) into the cells (2 × 10^6^) according to the recommended ratio. After transfection for 6 h, the medium was replaced with a complete medium, and the cells were cultured for an additional 24 h before further analysis.

### BEP‐Mediated Delivery Assay

≈2 × 10^6^ cells were mixed with 5 µg plasmid in 500 µL Opti‐MEM medium (31 985 070, ThermoFisher, USA). Electroporation was performed using the parameters of 30 V, 1 ms. Following electroporation, the cells were incubated in a complete medium for 24 h prior to further analysis.

### Cell Viability Assay

Cell viability was evaluated using the cell counting kit‐8 (CCK‐8) assay. Pre‐treated cells (≈1 × 10^4^ cells/well) were seeded into a 96‐well plate for 24 h. Subsequently, 10 µL CCK‐8 solution (CK18, Dojindo, China) was added to each well, followed by incubation for 1–4 h at 37 °C. The absorbance at 450 nm was then measured using a microplate reader (TECAN, Spark, Switzerland). Cell viability was calculated as the ratio of the absorbance of the experimental group to that of the control group.

### Delivery Efficiency Analysis

Following plasmid delivery, the cells were detached using 0.25% trypsin solution (G4013, Servicebio, China). The cell suspension was centrifuged at 500 × g for 5 min and washed twice with PBS. Finally, the cells were resuspended in 300 µL PBS and analyzed using a flow cytometer (DxFLEX, Beckman Coulter). The delivery efficiency was calculated as the percentage of GFP‐positive cells (expressing the GFP‐labeled plasmid) relative to the total cell population.

### Detection of EVs Yield

The total protein content of EVs secreted by an equal number of cells (1.5 × 10^7^) across all treatment groups was quantified using the BCA protein assay kit following the manufacturer's protocol, serving as the EV production for each treatment.

### Collection and Processing of Clinical Tumor Tissue Samples

Lung cancer tissue specimens were provided by Peking University Cancer Hospital & Institute. This study was approved by the Ethics Committee of Peking University Cancer Hospital & Institute (Institutional Review Board No. 2023KT146). The information of clinical patients that provided tumor tissue samples in Table S3 (Supporting Information). All sample donors signed informed consent forms. The excised tissues were preserved in a complete medium containing 10% FBS. After PBS rinsing to remove blood and impurities, the specimens were mechanically dissociated into small fragments (≈1 mm^3^ in diameter). These fragments were then digested in a 3 mL digestion solution, consisting of 2% FBS, 1 mg mL^−1^ collagenase I (C8140, Solarbio, China), and 200 µg mL^−1^ DNase I (D8071, Solarbio, China), at 37 °C for 2 h. The resulting suspension was filtered through a 70 µm cell strainer (352350, Corning, USA) and centrifuged at 350 × g for 10 min. Following PBS washing and repeat centrifugation, the cell pellet was resuspended in F‐12K medium supplemented with 15% FBS and FibrOut fibroblast inhibitor (4‐21564, CHI Scientific, China) in a cell incubator at 37 °C with 5% CO_2_.

### Collection and Processing of Clinical Blood Samples

Clinical blood samples were placed in blood collection tubes containing anticoagulants and diluted with PBS (blood: PBS = 1:1). Peripheral blood mononuclear cells (PBMCs) were isolated using human whole blood mononuclear cell separation solution (P9011, Solarbio, China). Briefly, the diluted blood was layered onto an equal volume of separation medium (half the volume of the diluted blood) and centrifuged at 800 × g for 20 min, with acceleration and deceleration rates set to 1. After centrifugation and washing with PBS, the PBMCs were preserved with cryopreservation solution (G1709, Servicebio, China) in liquid nitrogen.

### Animal Experiments

Female mice, including BALB/c nude (5–8 weeks), BALB/c (5–8 weeks), C57BL/6 (5–8 weeks), and NTG (4–5 weeks) mice, were obtained from SPF Laboratory Animal Technology in Beijing, China (Animal Certification No. SCXK 2019‐0010). Animals were kept in isolator cages within a pathogen‐free facility. All animal experiments were approved by the Laboratory Animal Ethics Committee of Beijing Cancer Hospital (Institutional Review Board No. 2023KT146) and conducted in compliance with the Guidelines on Humane Treatment of Laboratory Animals and standard procedures for animal care and use.

### In Vivo biodistribution of EVs

≈5 × 10^6^ tumor cells were subcutaneously injected into the flanks of BALB/c nude or BALB/c mice. One week later, EVs derived from tumor cells were labeled with DiD (V22887, ThermoFisher, USA) for 20 min and injected into mice via the tail vein. In vivo fluorescence imaging was performed using the IVIS Spectrum imaging system (Revvity, USA). Following imaging, the mice were euthanized, and tumors along with major organs were harvested for ex vivo imaging and frozen section.

### Tumor Metastasis Prevention Assay

In this study, both CDX and PDX tumor metastasis models were established. For the CDX model, BALB/c mice received subcutaneous injections of 5 × 10^6^ Lewis cells into the flank region. For the PDX model, NTG mice underwent surgical implantation of patient tumor fragments (2–3 mm^3^) into the inguinal region using hookless ophthalmic tweezers. Following two generations of tumor expansion (F1 and F2), the third generation (F3) tumor tissues were re‐transplanted into new NTG mice. Two weeks post‐engraftment, 7 × 10^6^ PBMCs were injected intraperitoneally to generate humanized mice with reconstituted immune systems. Immune reconstitution was verified through flow cytometric analysis of human CD45^+^ T cells in orbital blood. Meanwhile, Graft‐versus‐host disease (GVHD) was monitored twice weekly through clinical scores (based on weight, posture, activity, hair coat, skin, etc.). In both models, when tumors reached 40 mm^3^, visible tumor tissue was surgically excised and 2 × 10^5^ tumor cells were injected intravenously via the tail vein to simulate the scenario of tumor metastasis in vivo. Mice were then randomized into five treatment groups receiving different EVs preparations (100 µg per injection) via tail vein every two days for five total administrations. Following the treatment regimen, mice were euthanized for collection of blood, tumor tissues, and major organs to evaluate therapeutic efficacy.

### Tumor Metastasis Treatment Assay

≈5 × 10^6^ aggressive cells, selected through evolutionary screening on the SEE platform, were subcutaneously injected into BALB/c mice (CDX model) or NTG mice (PDX model). When tumors reached 80–100 mm^3^, mice were randomized into five treatment groups receiving different EVs preparations (100 µg per injection) via tail vein every two days for five total administrations. Following the treatment regimen, mice were euthanized for collection of blood, tumor tissues, and major organs to evaluate therapeutic efficacy.

### Histology and Immunohistochemistry Analysis

Tissue samples including heart, liver, spleen, lung, kidney, and tumor were fixed in 4% paraformaldehyde overnight, followed by paraffin embedding and frozen section at 10 µm thickness. For histological processing, all paraffin sections underwent dewaxing and hydration through sequential immersion in: 1) environmentally friendly dewaxing solution (G1128, Servicebio, China) I 10 min, 2) environmentally friendly dewaxing solution II 10 min, 3) environmentally friendly dewaxing solution III 10 min, 4) anhydrous ethanol I 5 min, 5) anhydrous ethanol II 5 min, and 6) anhydrous ethanol III 5 min, with a final rinse in tap water. Subsequently, the sections were subjected to hematoxylin‐eosin (HE) staining, Ki67 immunohistochemistry, and TUNEL apoptosis assay.

### HE Staining

HE staining was performed on dewaxed sections using a hematoxylin‐eosin staining kit (G1076, Servicebio, China) according to the manufacturer's protocol. The sections underwent sequential steps, including dewaxing, staining pretreatment, hematoxylin staining, eosin staining, dehydration, and sealing. After processing, images were acquired and analyzed, with nuclei staining blue and cytoplasm staining red.

### Ki67 Staining

Dewaxed sections were processed through the following sequential steps: antigen recovery, endogenous peroxidase blocking, non‐specific site blocking, primary antibody incubation (GB121141, Servicebio, China), and secondary antibody incubation (GB23301, Servicebio, China). Following PBS washes, sections were treated with freshly prepared DAB chromogenic solution (G1212, Servicebio, China). The reaction was terminated by rinsing with tap water. Subsequently, sections were counterstained with hematoxylin, dehydrated, and mounted. Immunostaining results were evaluated under microscopy.

### TUNEL Assay

Dewaxed sections were processed using a TUNEL staining kit (G1504, Servicebio, China) following the manufacturer's protocol. Briefly, slides were sequentially treated with protease K for antigen retrieval, permeabilized, equilibrated at room temperature, and incubated with the reaction mixture (TDT enzyme: dUTP: buffer = 1: 5: 50). After nuclear counterstaining with DAPI, the sections were mounted for fluorescence microscopy imaging.

### Kidney and Liver Function Analysis

Mouse peripheral blood was collected via orbital bleeding. Following a 2 h standing at room temperature, the samples were centrifuged at 1500 × g for 15 min. The supernatant was subsequently analyzed using an automated biochemical analyzer.

### Flow Cytometry Assay

The cells were suspended in 100 µL cell staining buffer (E‐CK‐A107, Elabscience, China) and incubated with an anti‐CD16 antibody for 10 min. Subsequently, specific antibodies for T cells and macrophages (details are provided in Table S4, Supporting Information) were added for cell identification. Following 30 min incubation, the cells were added into 1 mL cell staining buffer and centrifuged at 350 × g for 10 min at 4 °C. The cells were then resuspended in a fixation buffer (E‐CK‐A109, Elabscience, China) for 30 min. After washes with cell staining buffer, the cells were analyzed using flow cytometry (LSRFORTESSA, Becton, Dickinson and Company, America).

### Statistical Analysis

To simulate the distribution of the electric field, a simplified model of the electro‐remodeling module was established by using COMSOL Multiphysics (version 6.1). The data were analyzed using GraphPad Prism 8.0, Origin 8.0, Flowjo 10.8, and Image J FIJI software. Student's *t*‐test and one‐way ANOVA were used for comparisons between groups. The data were presented as mean ± standard deviation (SD). Differences were considered statistically significant when *p* < 0.05.

## Conflict of Interest

The authors declare no conflict of interest.

## Supporting information



Supporting Information

## Data Availability

All data are available in the main text or the supplementary materials.
