# Supplementary material for: A Nano‐Electro‐Platform Enabling Evolutionary Screening and Remodeling of Tumor Cells for Metastasis Inhibition
Source: Adv Sci (Weinh). 2025 Jun 20;12(34):e07684. doi: 10.1002/advs.202507684 (PMC12442665; doi:10.1002/advs.202507684)
Supplement: Supplementary file 1 — Supporting Information [file ADVS-12-e07684-s001.docx]

Supporting Information

A nano-electro-platform enabling evolutionary screening and remodeling of tumor cells for metastasis inhibition

Feng Liu, Hong Sun, Bing Liu, Jing Zhang, Chengbao Wu, Shi Yan, Chengzheng Tai, Yihang Tong, Rongtai Su, Xiaowei Xiang, Han Wu, Fuqi Yao, Kuan Yang, Dedong Yin, Yuqiong Wang, Ao Xiao, Long Cheng, Xi Chen*, Nan Wu*, Zaizai Dong*, Lingqian Chang*

The file includes:

Supplemental Figures

Supplemental Tables


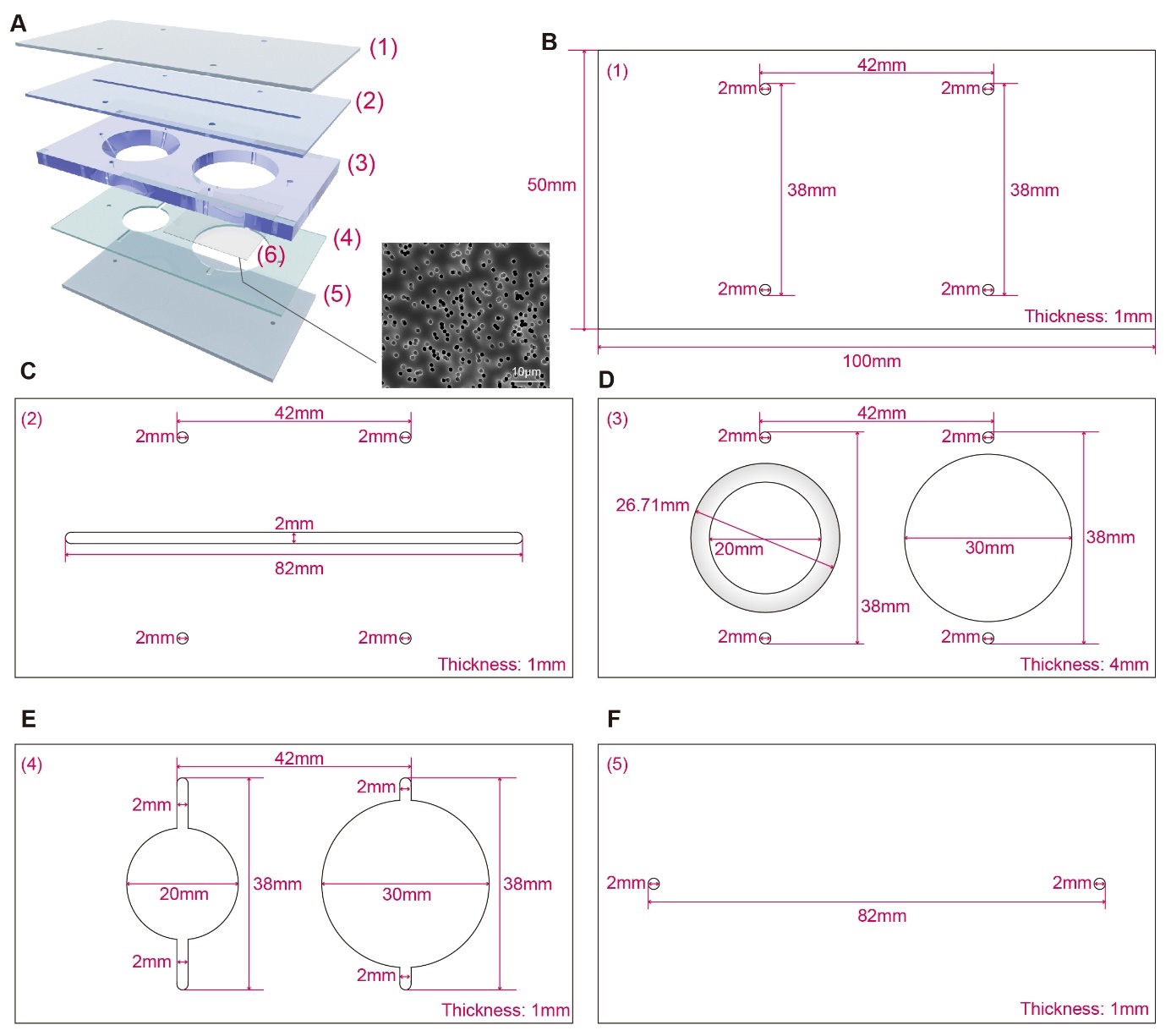


Figure S1. The design and dimensional schematic of the SEE platform. A) The exploded view of the SEE platform revealed its functional layers, which were: (1) sealing layer; (2) microchannel layer; (3) cell culture layer for evolutionary screening (left) and electrical remodeling (right); (4) cell culture chamber (left) and delivery cargo storage chamber (right); (5) electrode layer; and (6) nanomembrane. And here, the TEM image of the nanomembrane was presented (scale bars = 10 μm). B-F, Dimensions of the functional layer of (1)-(5), respectively.

**
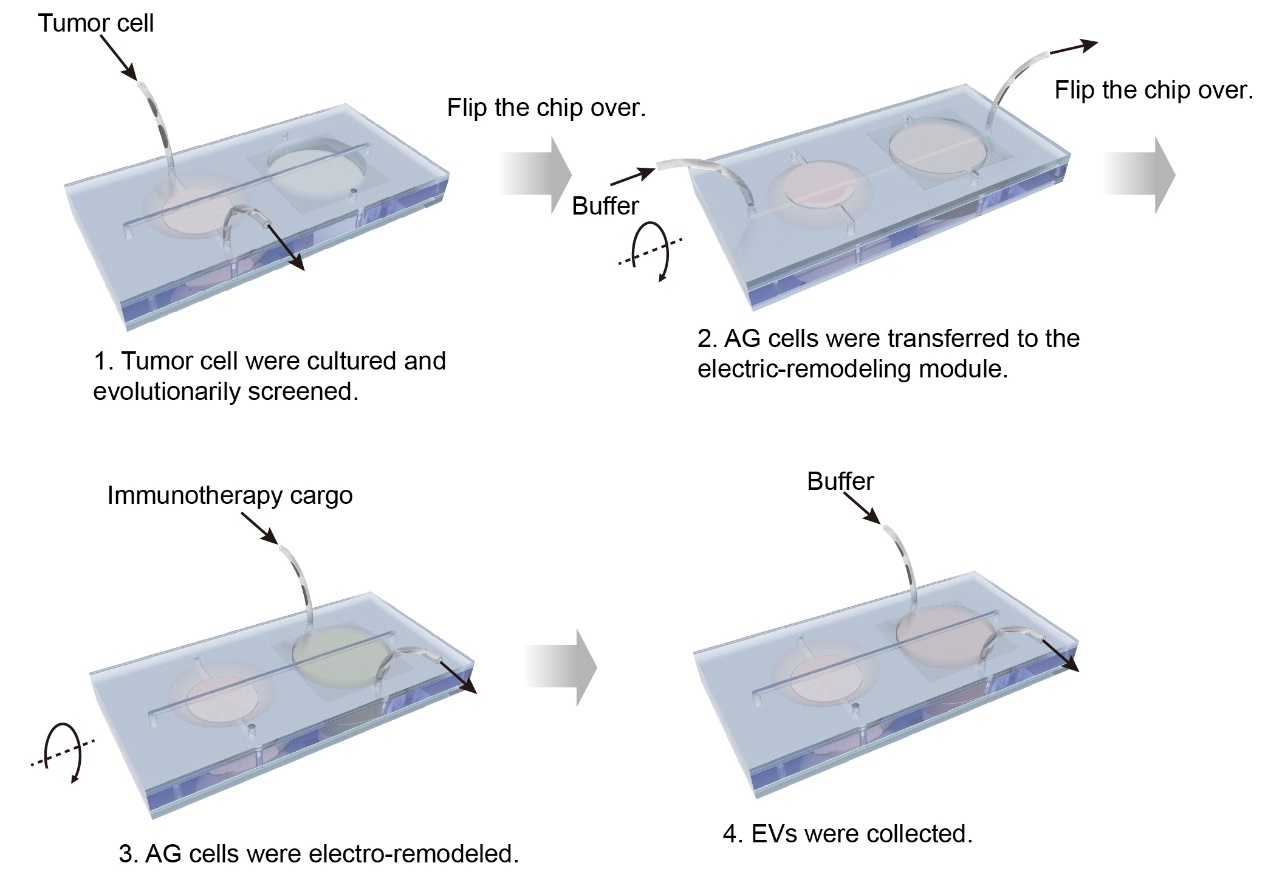
**

**Figure** **S2.** Workflow of the SEE Platform. As shown in steps 1-4 of the diagram.


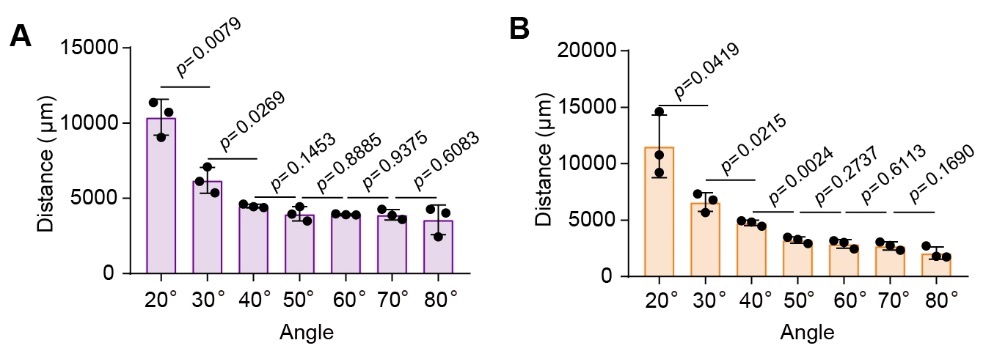


**Figure** **S3.** Selection of evolutionary screening slope. A, B) Quantitative analysis of the evolutionary screening slope by A549 (A) and H1975 cells (B). The data were derived from three independent experiments and were presented as mean ± SD; two-sided Student’s *t*-test was used for comparisons (A, B). *p* < 0.05 was considered statistically significant.


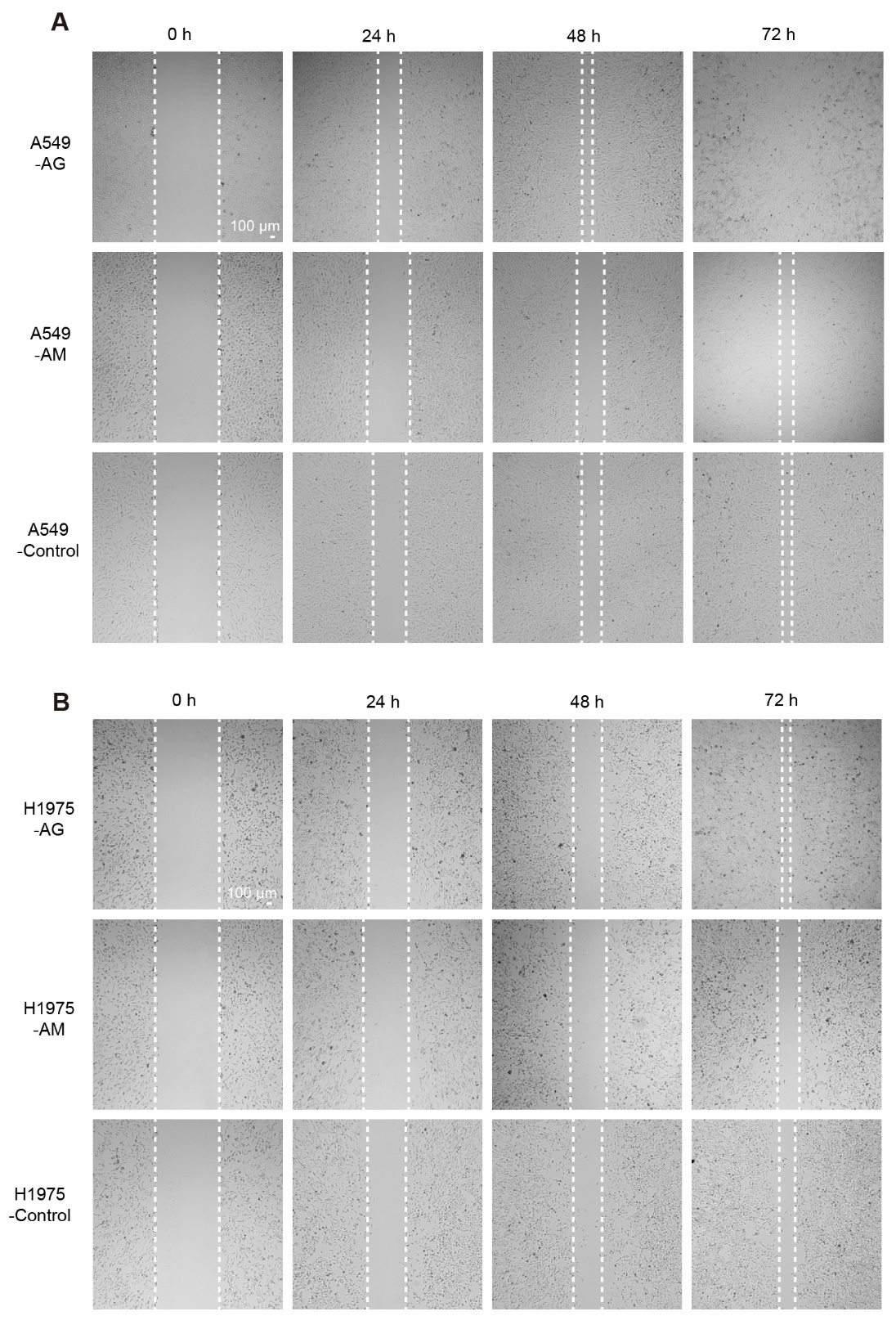


**Figure S4.** Images of cell wound healing assay. A, B) The migration ability of A549 (A) and H1975 cells (B) (scale bars = 100 μm). Cell-AG, Aggressive cell; Cell-AM, Ambitionless cell; Cell-Control, Normal cell.


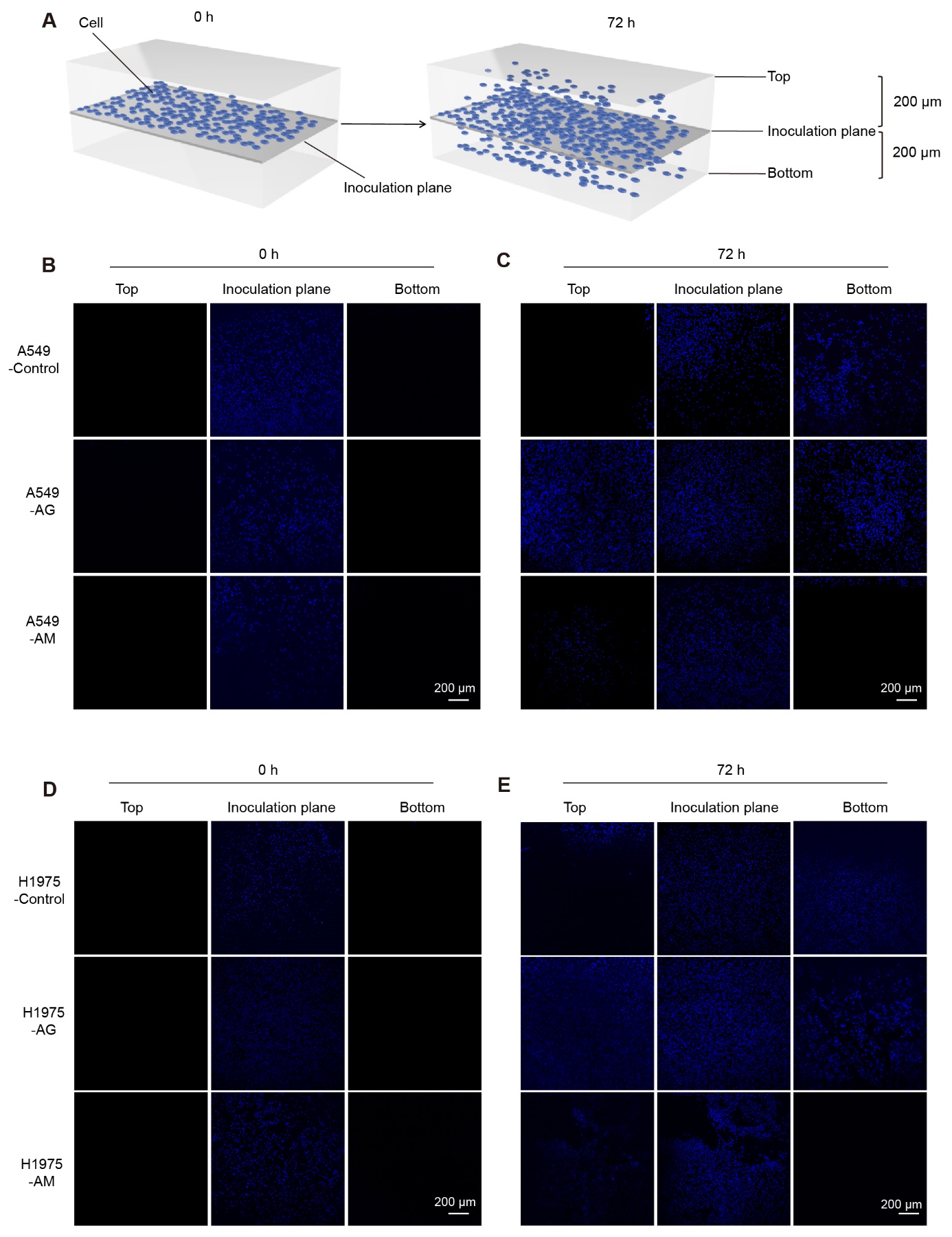


**Figure** **S5.** Characterization of cell invasion capability in three dimensions. A) Schematic diagram of 3D invasion assay. B, C) Images of A549 cells obtained through evolutionary screening at different Z-plane positions (scale bars = 200 μm). D, E) Images of H1975 cells obtained through evolutionary screening at different Z-plane positions (scale bars = 200 μm).


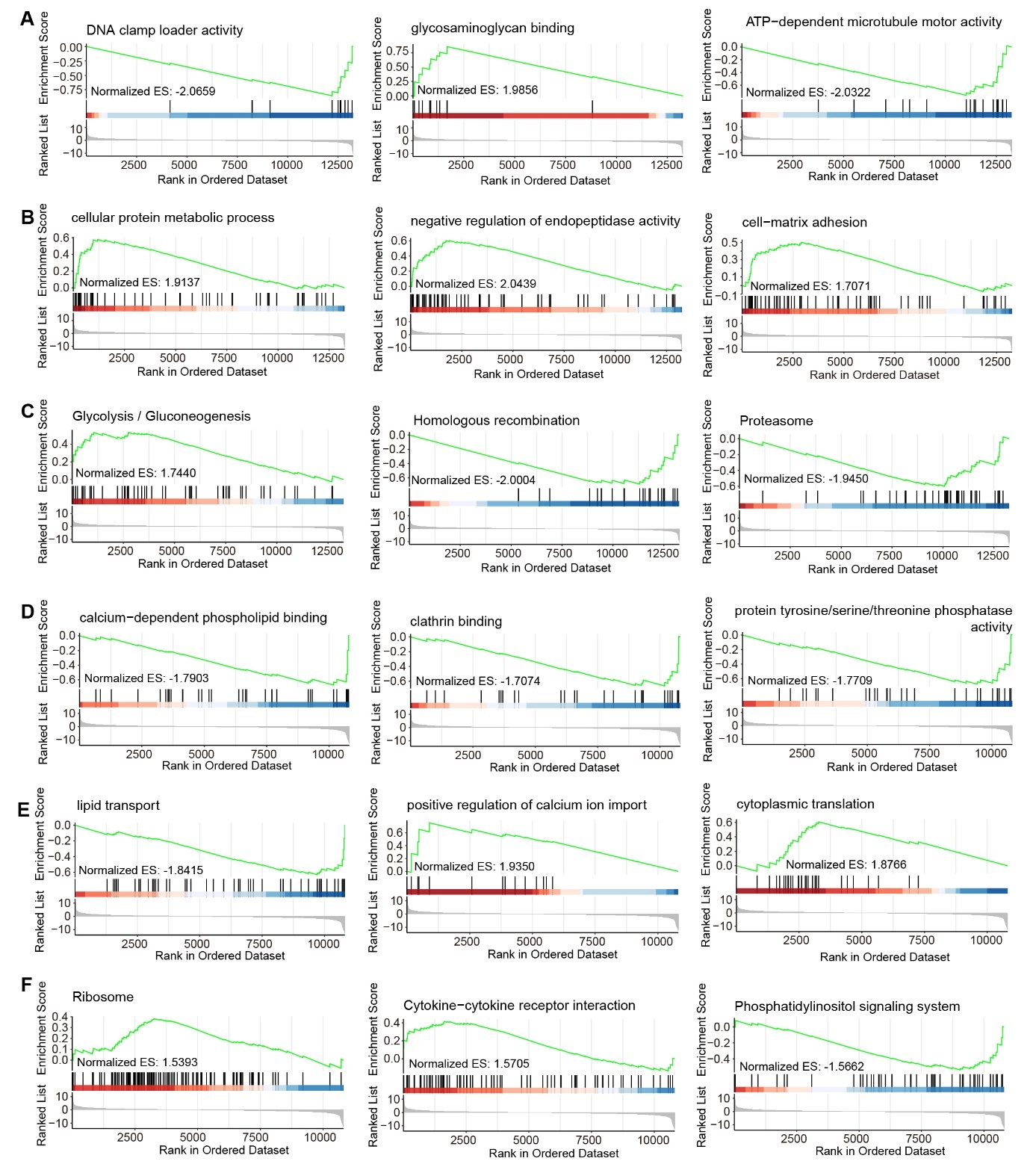


**Figure** **S6.** Gene set enrichment analysis. A-C) Variant genes between A549-AG and A549-AM cells in molecular function (A), biological processes (B) and KEGG pathway (C). D-F) Variant genes between H1975-AG and H1975-AM cells in molecular function (D), biological processes (E) and KEGG pathway (F). The GSEA enrichment results exhibited the TOP3 genes.


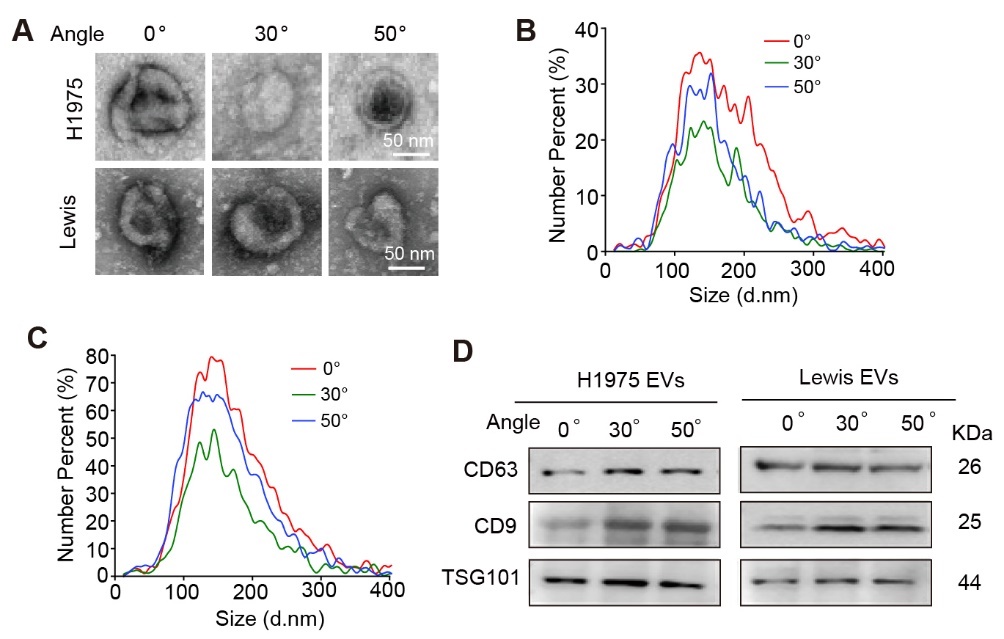


**Figure** **S7.** Identification of EVs derived from cells screened on different slopes. A) TEM images of EVs derived from H1975 (top) and Lewis cells (bottom) (scale bars = 50 nm). 0°, 30° and 50° represented EVs derived from cells screened at the angle of 0°, 30° and 50°, respectively. B, C) NTA analysis of EVs derived from H1975 (B) and Lewis cells (C). D) Western blot analysis of markers (CD63, CD9, and TSG101) on EVs derived from H1975 (left) and Lewis cells (right).


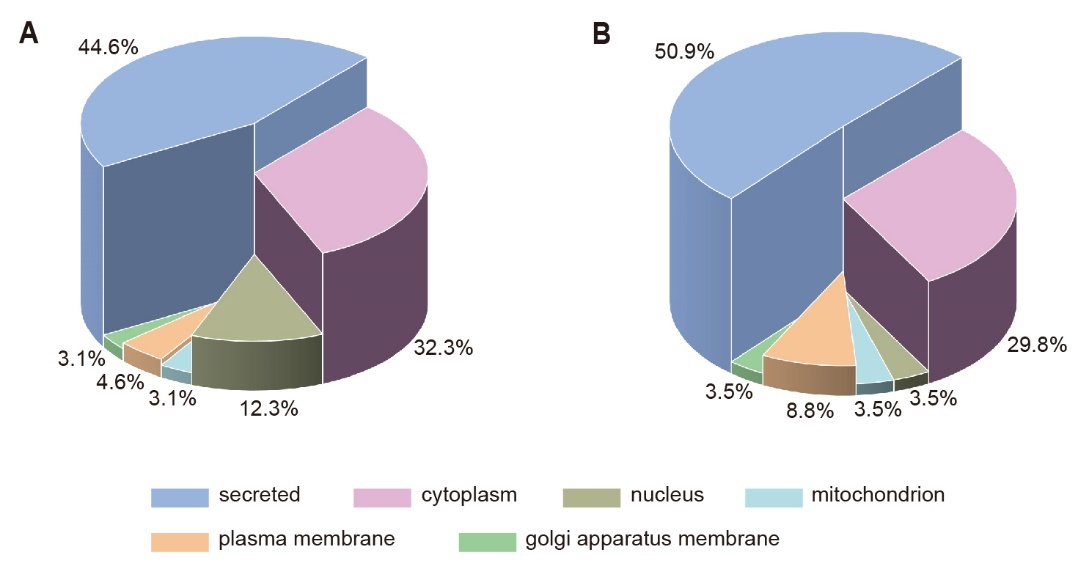


**Figure** **S8.** Subcellular localization analysis of differentially expressed proteins in EVs derived from cells after evolutionary screening. A) The differential proteins between EVs@A549-AM and EVs@A549-AG. B) The differential proteins between EVs@H1975-AM and EVs@H1975-AG.


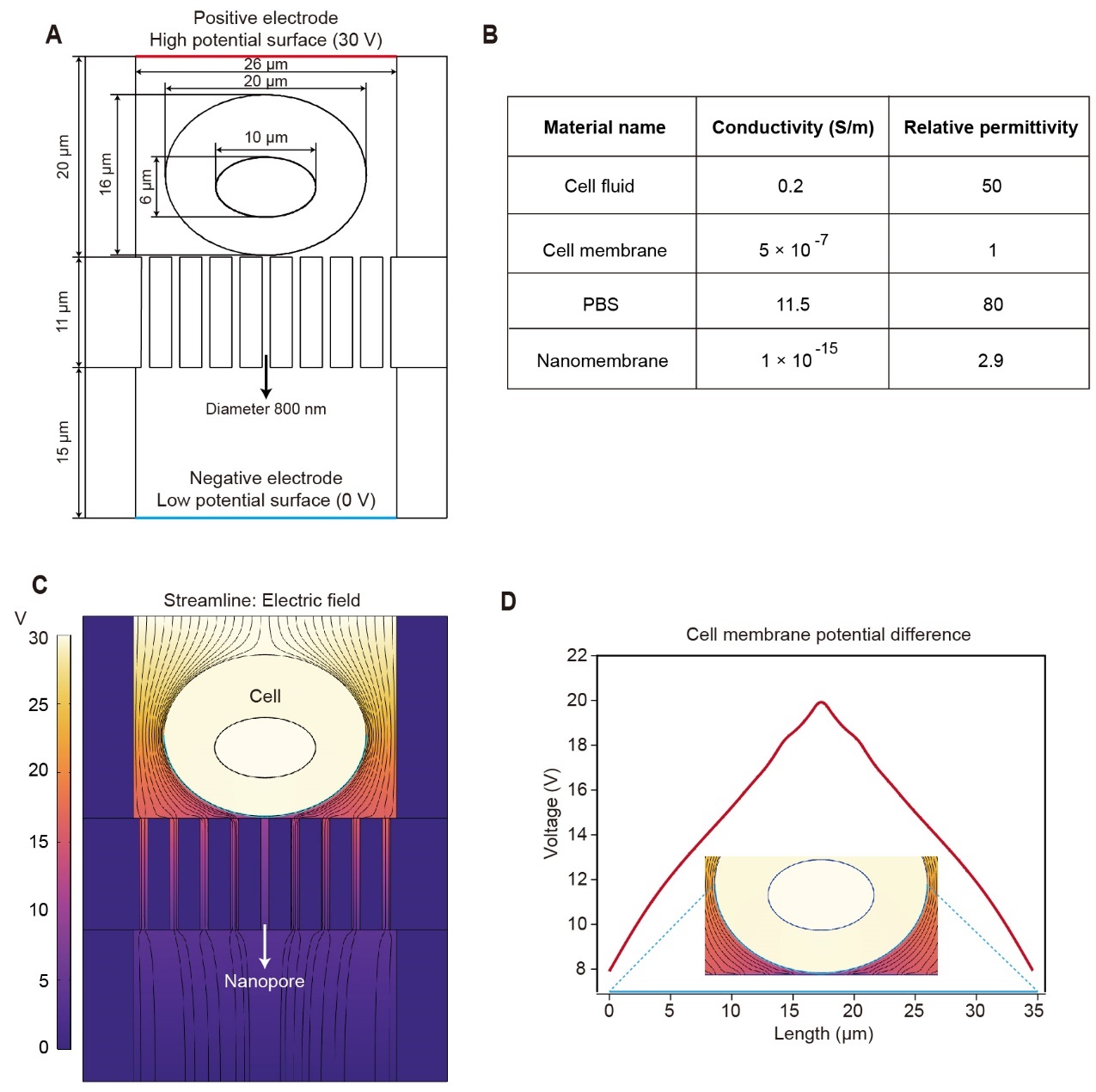


**Figure** **S9.** Electric field simulation of the electric-remodeling module on the SEE platform. A) Simplified dimensional parameters for the simulation of electric field. B) Parameters of the materials used in the simulation. C) Distribution of the electric potential around the cell. D) The electrical potential difference between the inside and outside of the cell membrane measured at the blue circle every 0.1 µm. Length represents the flattened arc length of the lower edge of the cell.


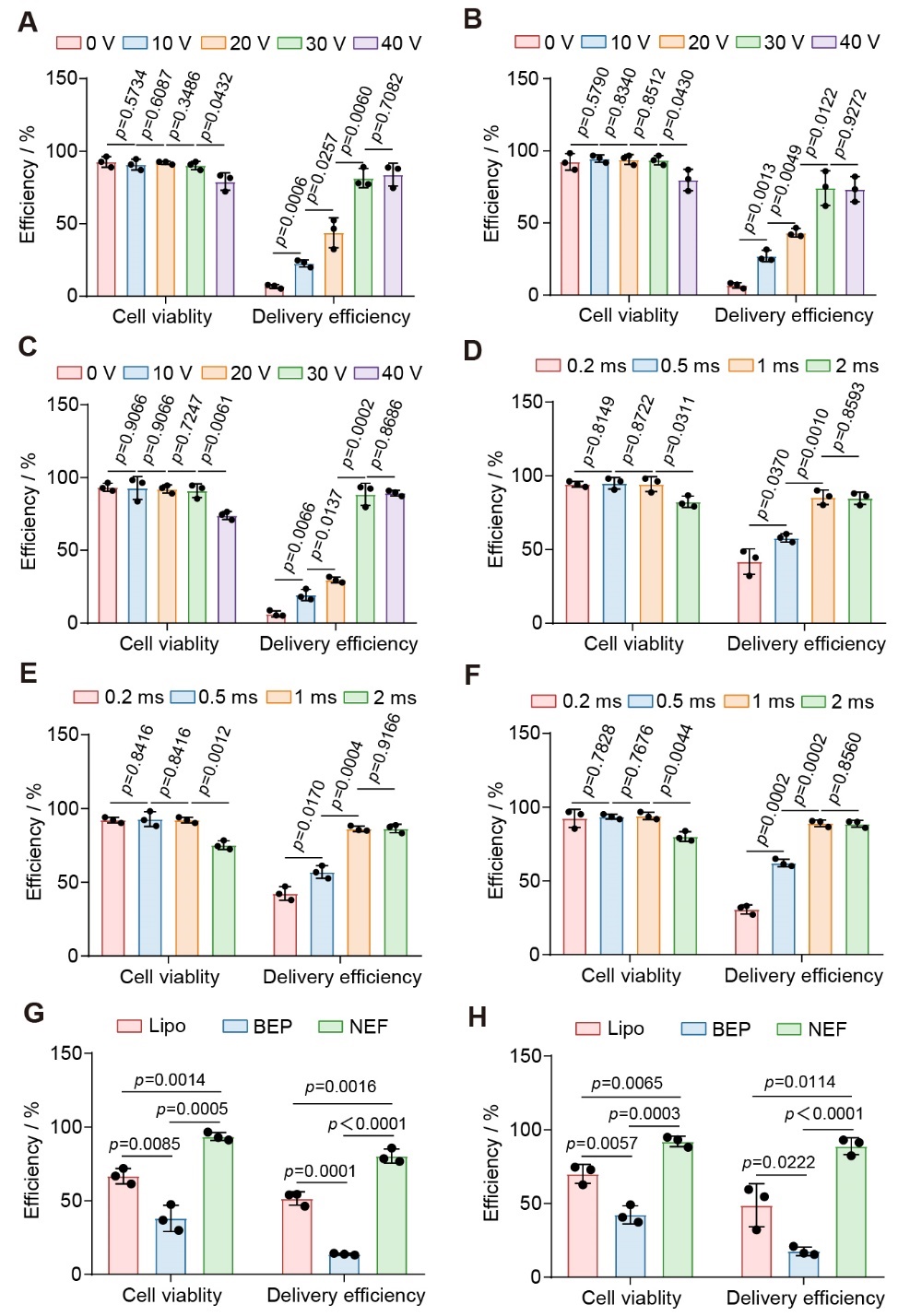


**Figure** **S10.** Comparison of different treatments in terms of cell viability and delivery efficiency. A-C) Cell viability and delivery efficiency in A549 (A), H1975 (B) and Lewis cells (C) following the application of different voltages (0 V, 10 V, 20 V, 30 V, 40 V), respectively. D-F) Cell viability and delivery efficiency in A549 (D), H1975 (E) and Lewis cells (F) following the application of different pulse durations (0.2 ms, 0.5 ms, 1 ms, 2 ms), respectively. G, H) Cell viability and delivery efficiency after delivery of H1975 (G) and Lewis cells (H) using Lipo, BEP and NEF, respectively. The data were derived from three independent experiments and were presented as mean ± SD; two-sided Student’s *t*-test was used for comparisons (A-H). *p* < 0.05 was considered statistically significant.


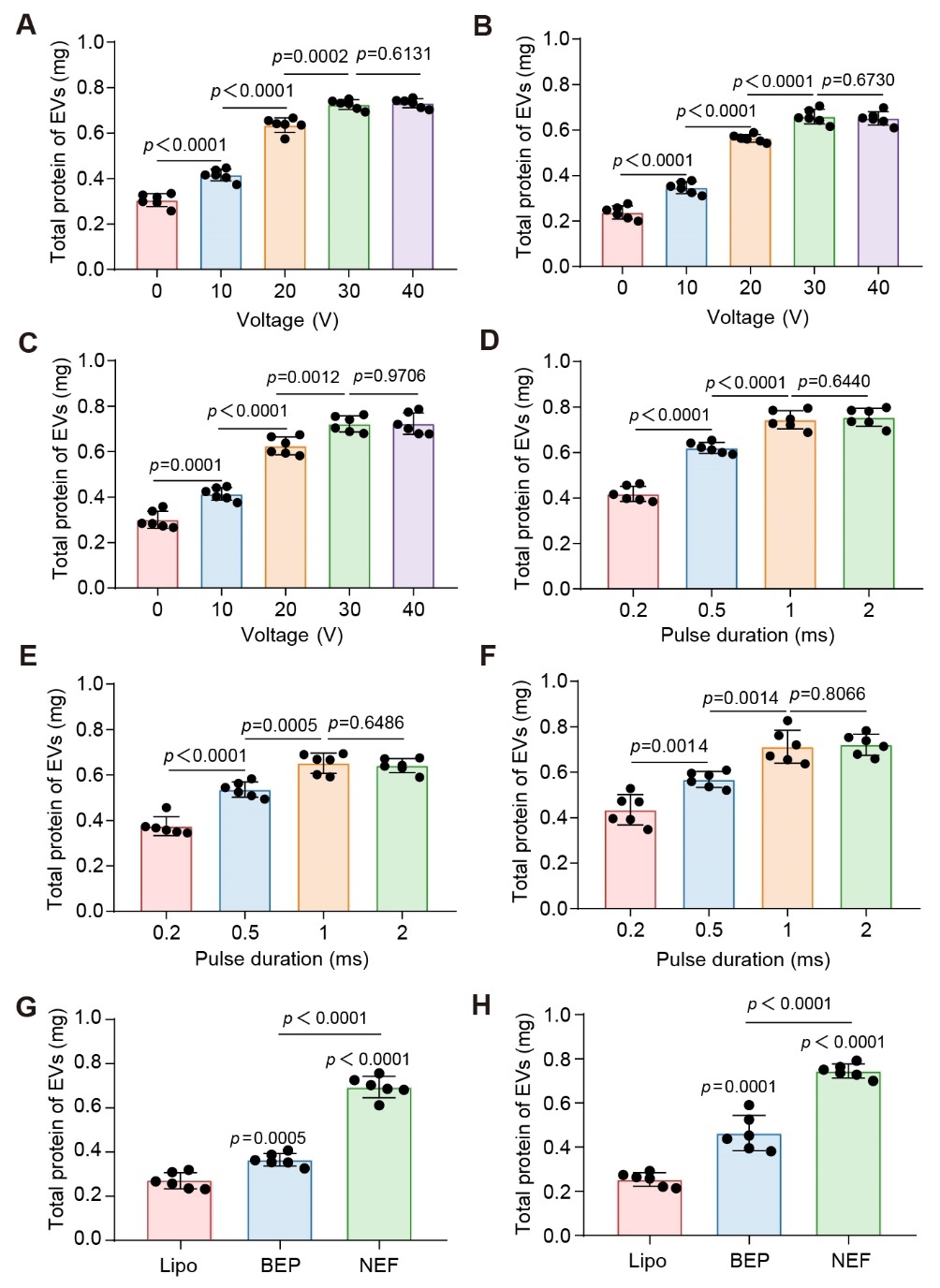


**Figure** **S11.** EVs yield under different cell treatments. A-C) Total protein of EVs produced by A549 (A), H1975 (B) and Lewis cells (C) with applying different voltages (0 V, 10 V, 20 V, 30 V, 40 V), respectively. D-F) Total protein of EVs produced by A549 (D), H1975 (E) and Lewis cells (F) with applying different pulse durations (0.2 ms, 0.5 ms, 1 ms, 2 ms), respectively. G, H) Total protein of EVs produced by H1975 (G) and Lewis cells (H) after treating with Lipo, BEP and NEF, respectively. The data were derived from six independent experiments and were presented as mean ± SD; two-sided Student’s *t*-test was used for comparisons (A-H). *p* < 0.05 was considered statistically significant.


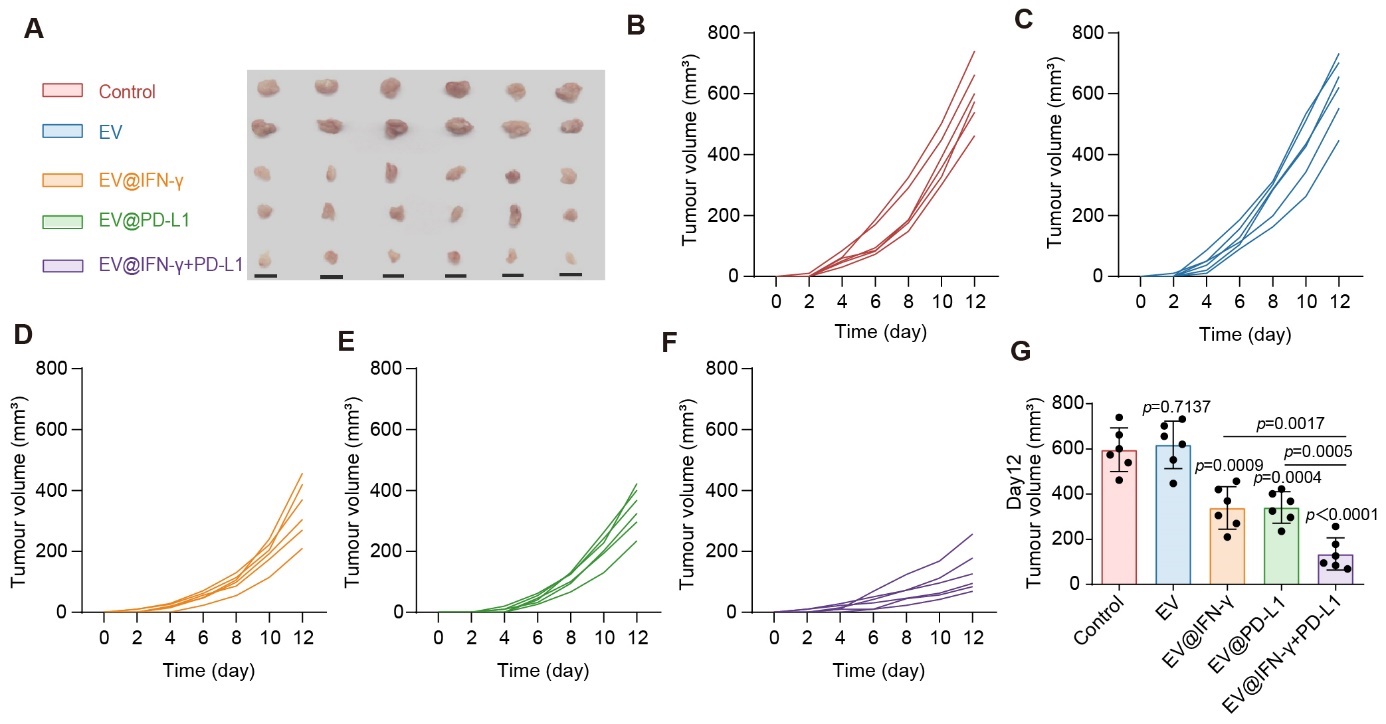


**Figure** **S12.** The therapeutic efficacy of SEE-EVs in CDX model for preventing tumor metastasis. A) Images showing the tumor size at the end of treatment. B-F) Tumor growth curves of the CDX model mice during the course of treatment. Control (B), EV (C), EV@IFN-γ (D), EV@PD-L1 (E), and EV@IFN-γ+PD-L1 (F). G) Statistical analysis of tumor volumes in each group at the end of treatment. The data were derived from six independent experiments and were presented as mean ± SD; two-sided Student’s *t*-test was used for comparisons (G). *p* < 0.05 was considered statistically significant.


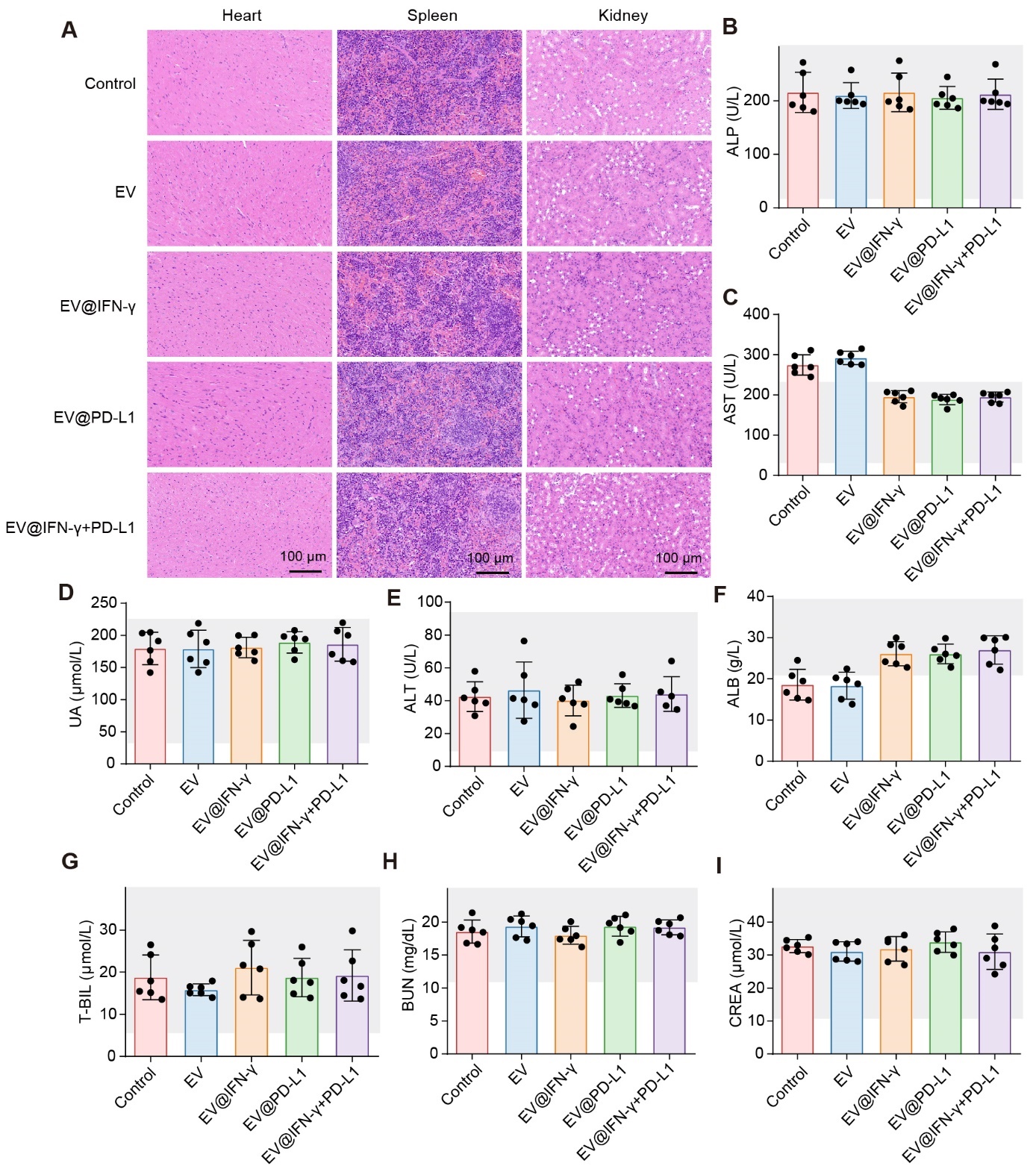


**Figure** **S13.** Evaluation of SEE-EVs in tumor metastatic inhibition in CDX model from a biosecurity perspective. A) H&E staining of main organs in different groups at the end of treatment (scale bar = 100 μm) (n = 3 mice per group). B-I) Serum biochemistry parameters of different groups at the end of treatment. ALP, Alkaline phosphatase (B); AST, Aspartate aminotransferase (C); UA, Uric acid (D); ALT, Alanine aminotransferase (E); ALB, Albumin (F); T-BIL, Total bilirubin (G); BUN, Blood urea nitrogen (H); CREA, Serum creatinine(I). n = 6 mice per group. The gray background represented the normal range of each parameter.


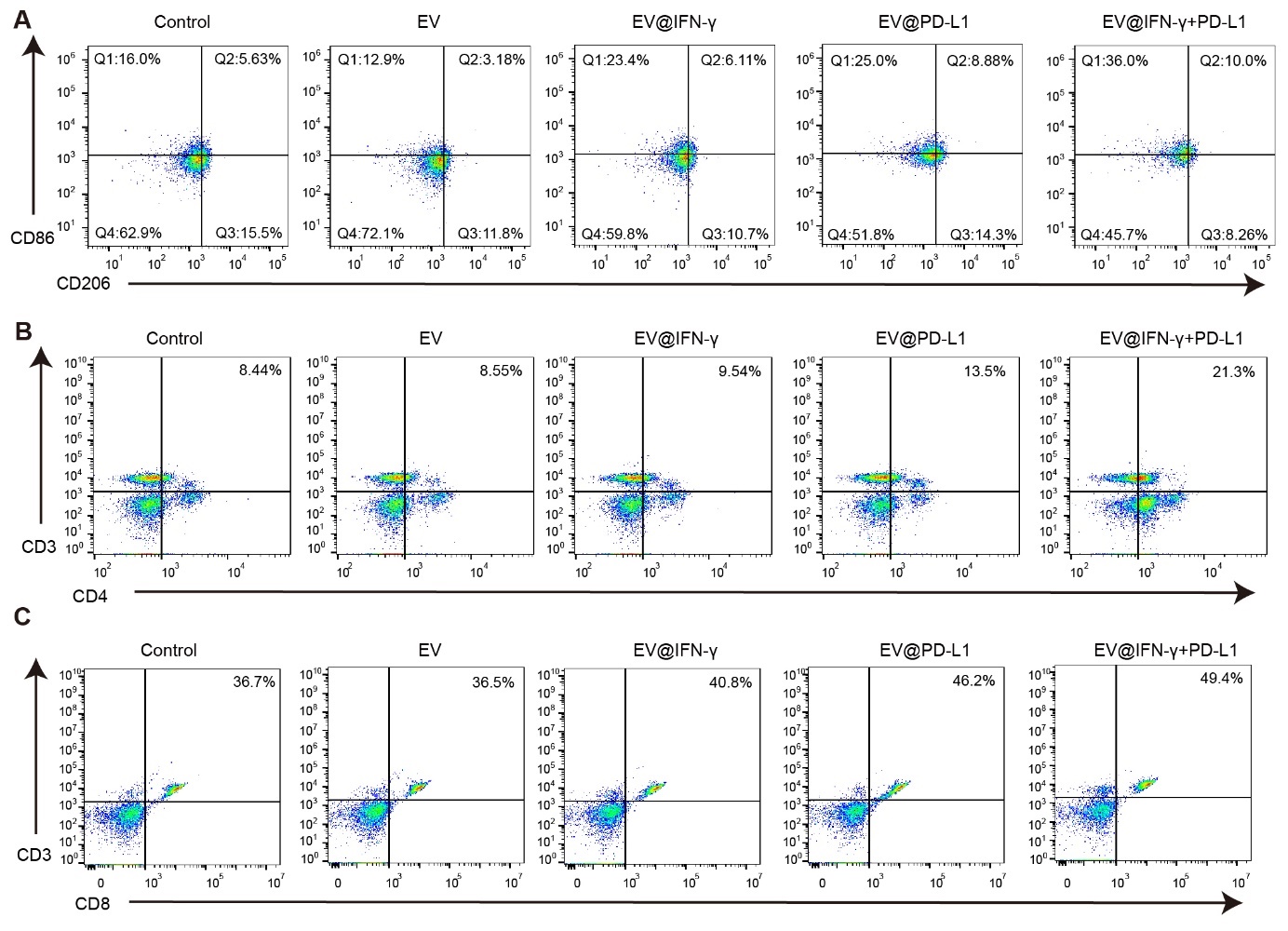


**Figure S14.** Characterization of macrophage phenotypes and activation of T cells in blood after treatment with SEE-EVs in CDX model. A) FACS scatter plot analysis of the expression of CD86 (marker of M1 macrophages) and CD206 (marker of M2 macrophages). B, C) FACS scatter plot analysis of the infiltration of CD4^+^ T cells (B) and CD8^+^ T cells (C). CD3 was a marker to identify T cells. The data were from six independent experiments and were presented as mean ± SD.


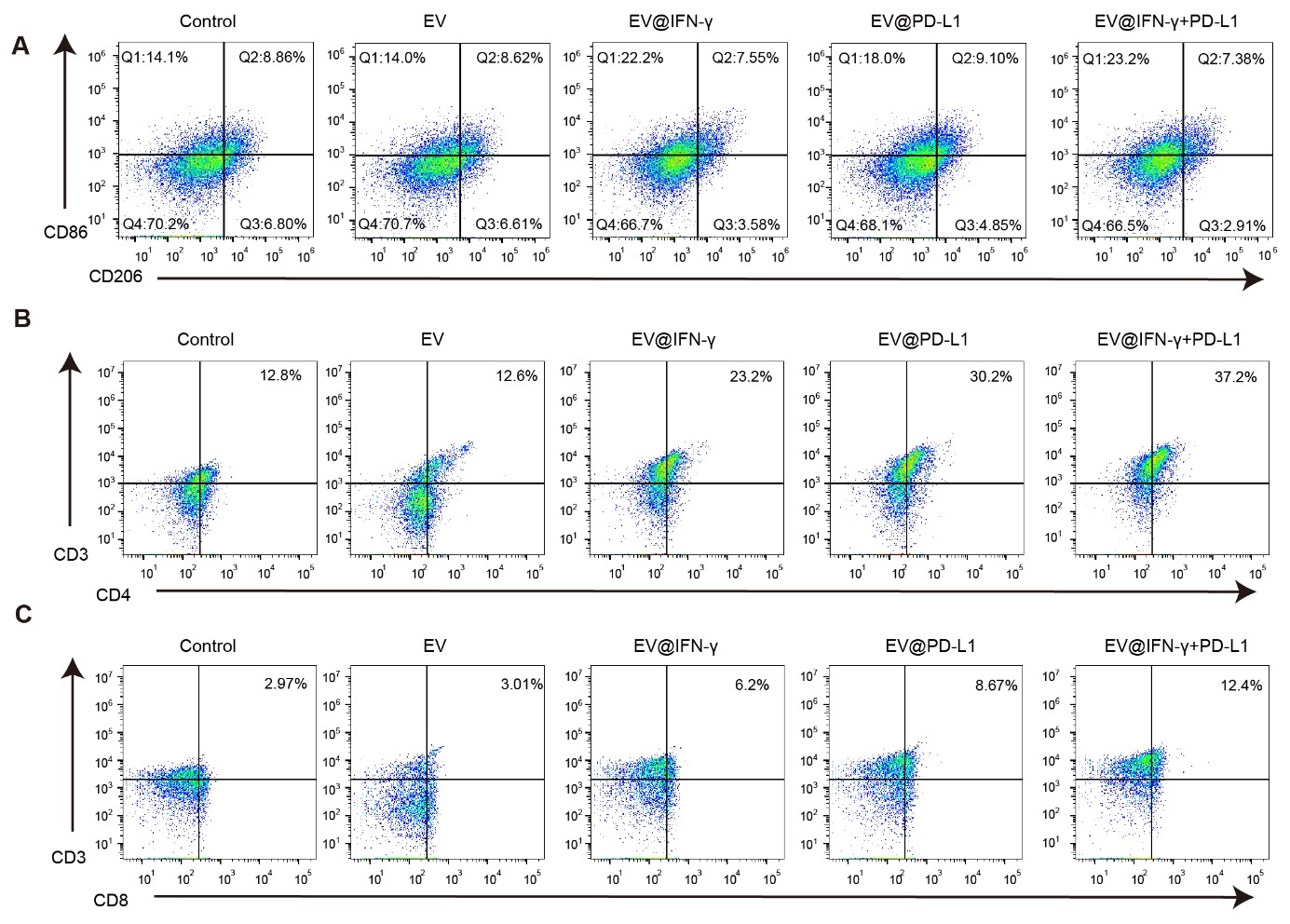


**Figure** **S15.** Characterization of macrophage phenotypes and activation of T cells in tumor tissues after treatment with SEE-EVs in CDX model. A) FACS scatter plot analysis of the expression of CD86 (marker of M1 macrophages) and CD206 (marker of M2 macrophages). B, C) FACS scatter plot analysis of the infiltration of CD4^+^ T cells (B) and CD8^+^ T cells (C). The data were from six independent experiments and were presented as mean ± SD.


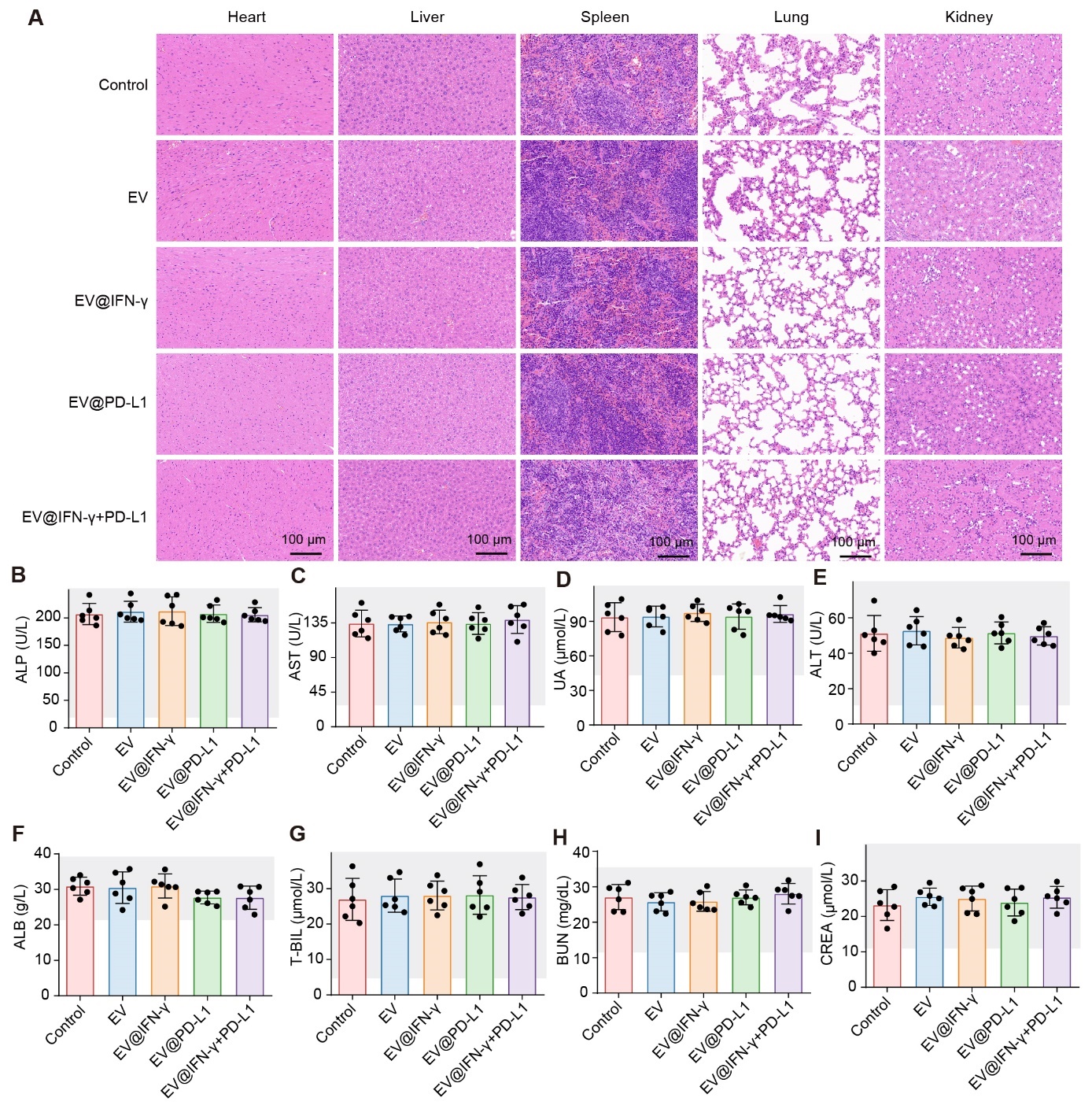


**Figure** **S16.** Biosecurity assessment of SEE-EVs in CDX model. A) H&E staining of main organs in different groups at the end of treatment (scale bar = 100 μm) (n = 3 mice per group). B-I) Serum biochemistry parameters of different groups at the end of treatment, including ALP (B), AST (C), UA (D), ALT (E), ALB (F), T-BIL (G), BUN (H), and CREA (I). n = 6 mice per group. The gray background represented the normal range of each parameter.


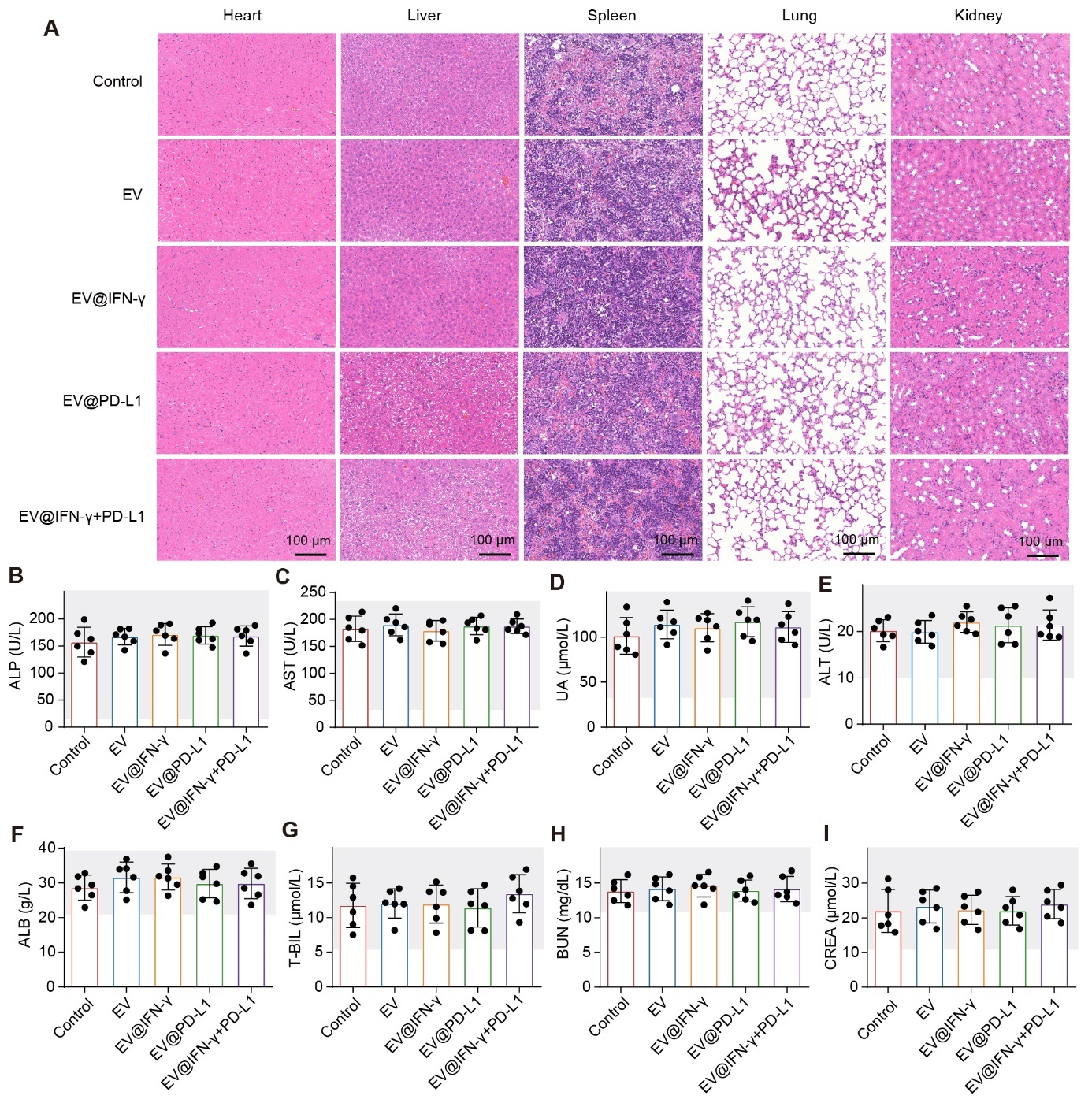


**Figure** **S17.** Biosecurity assessment of SEE-EVs in PDX model. A) H&E staining of main organs in different groups at the end of treatment (scale bar = 100 μm) (n = 3 mice per group). B-I) Serum biochemistry parameters of different groups at the end of treatment, including ALP (B), AST (C), UA (D), ALT (E), ALB (F), T-BIL (G), BUN (H), and CREA (I). n = 6 mice per group. The gray background represented the normal range of each parameter.


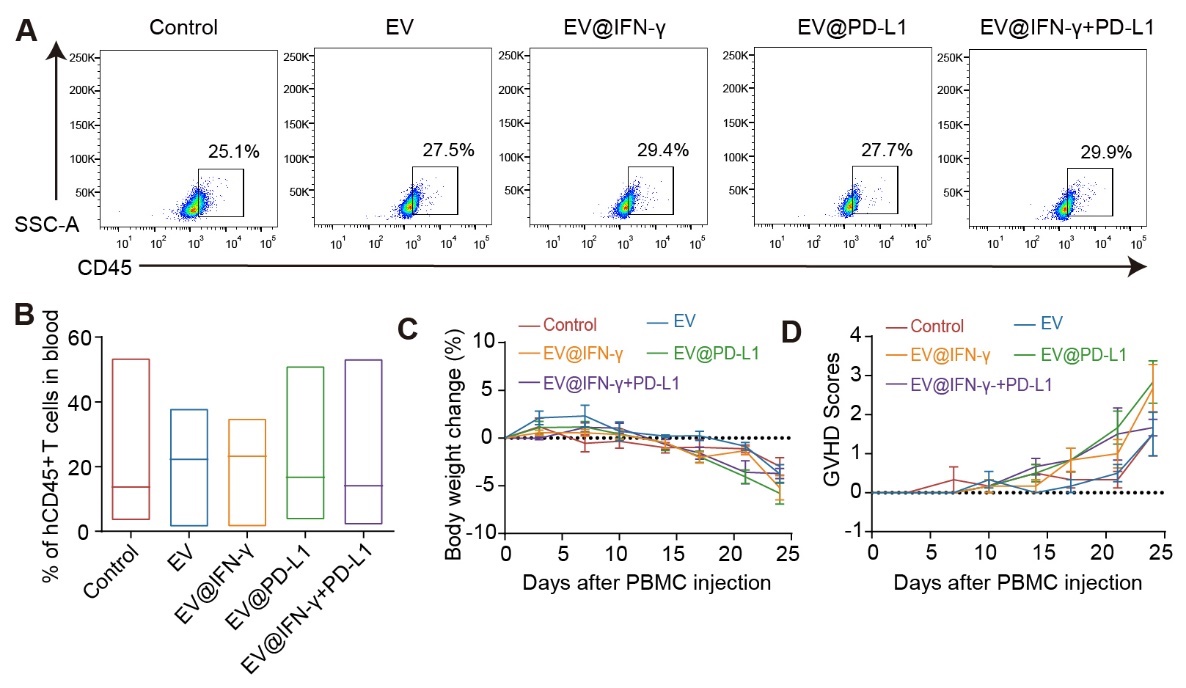


**Figure** **S18.** Reconstruction of human immune system. A) FACS analysis of the expression of CD45^+^ T cells in blood. B) Quantitative analysis of CD45^+^ T cells in blood for each mouse corresponding to (A). C) Weight change in humanized mice. D) Disease score in humanized mice. Graft-versus-host disease, GVHD. n = 6 mice per group.


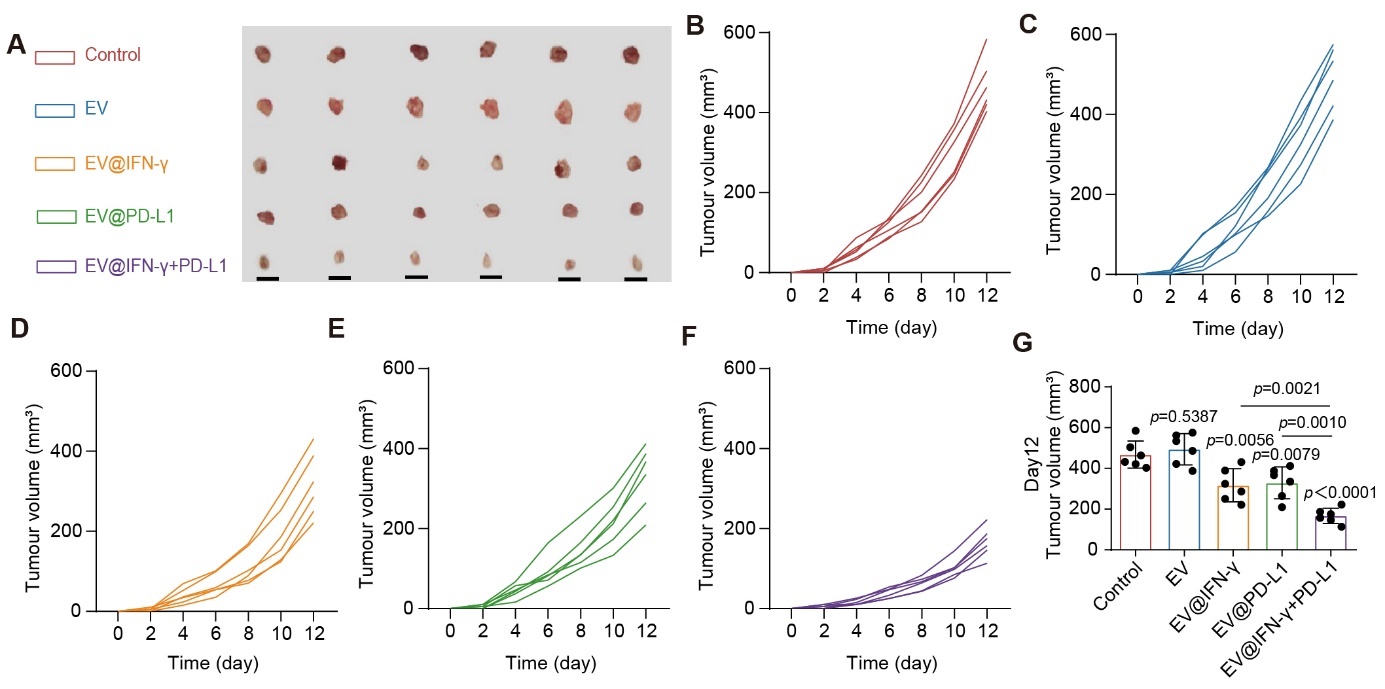


**Figure S19.** The therapeutic efficacy of SEE-EVs in PDX model for preventing tumor metastasis. A) Images showing the tumor size at the end of treatments. B-F) Tumor growth curves of the PDX model mice during the course of treatment. Control (B), EV (C), EV@IFN-γ (D), EV@PD-L1 (E), and EV@IFN-γ+PD-L1 (F). G) Statistical analysis of the tumor volumes in each group at the end of treatment. The data were derived from six independent experiments and were presented as mean ± SD; two-sided Student’s *t*-test was used for comparisons (G). *p* < 0.05 was considered statistically significant.


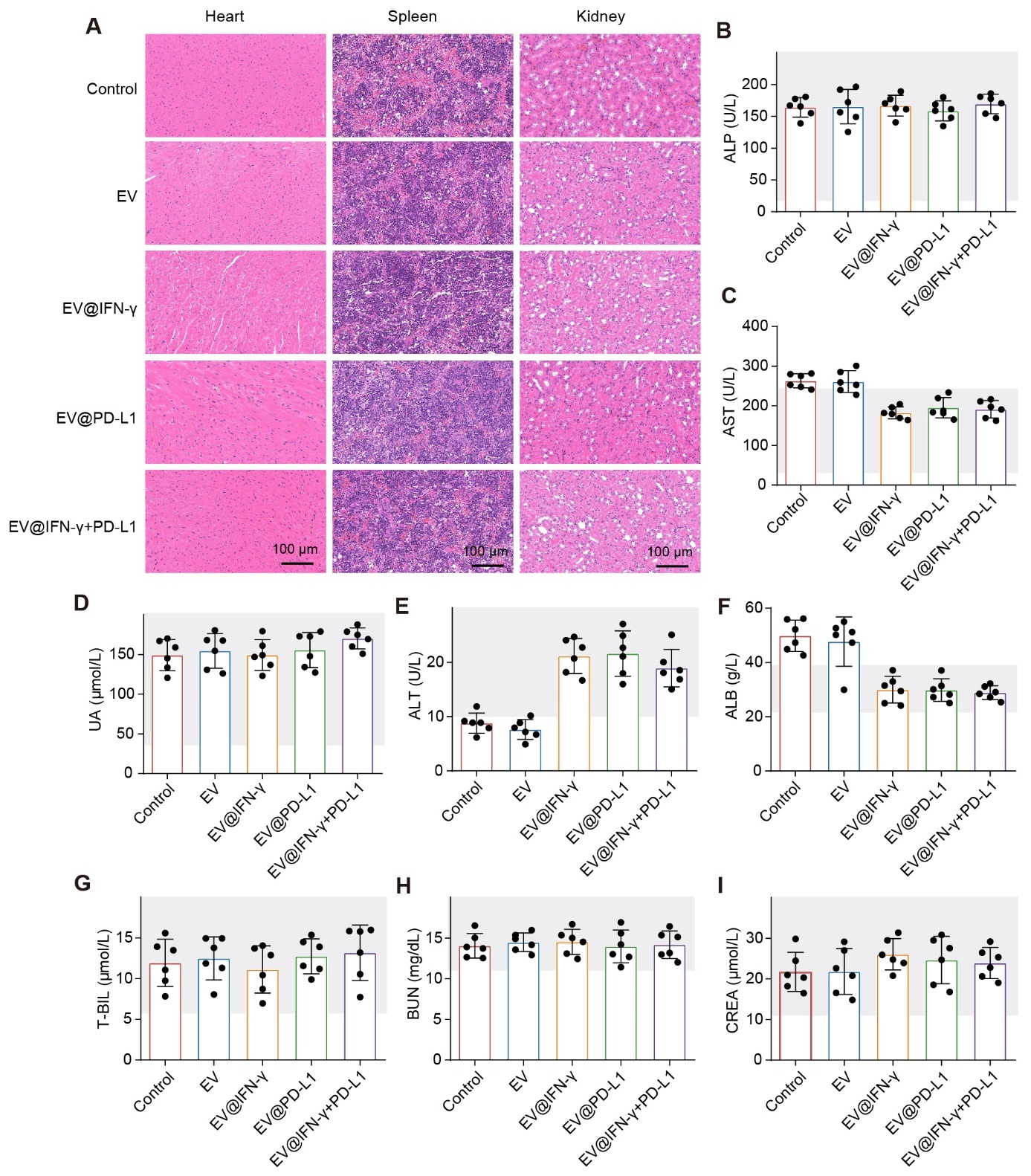


**Figure** **S20.** Evaluation of SEE-EVs in tumor metastatic inhibition in PDX model from a biosecurity perspective. A) H&E staining of main organs in different groups at the end of treatment (scale bar = 100 μm) (n = 3 mice per group). B-I) Serum biochemistry parameters of different groups at the end of treatment, including ALP (B), AST (C), UA (D), ALT (E), ALB (F), T-BIL (G), BUN (H), and CREA (I). n = 6 mice per group. The gray background represented the normal range of each parameter.


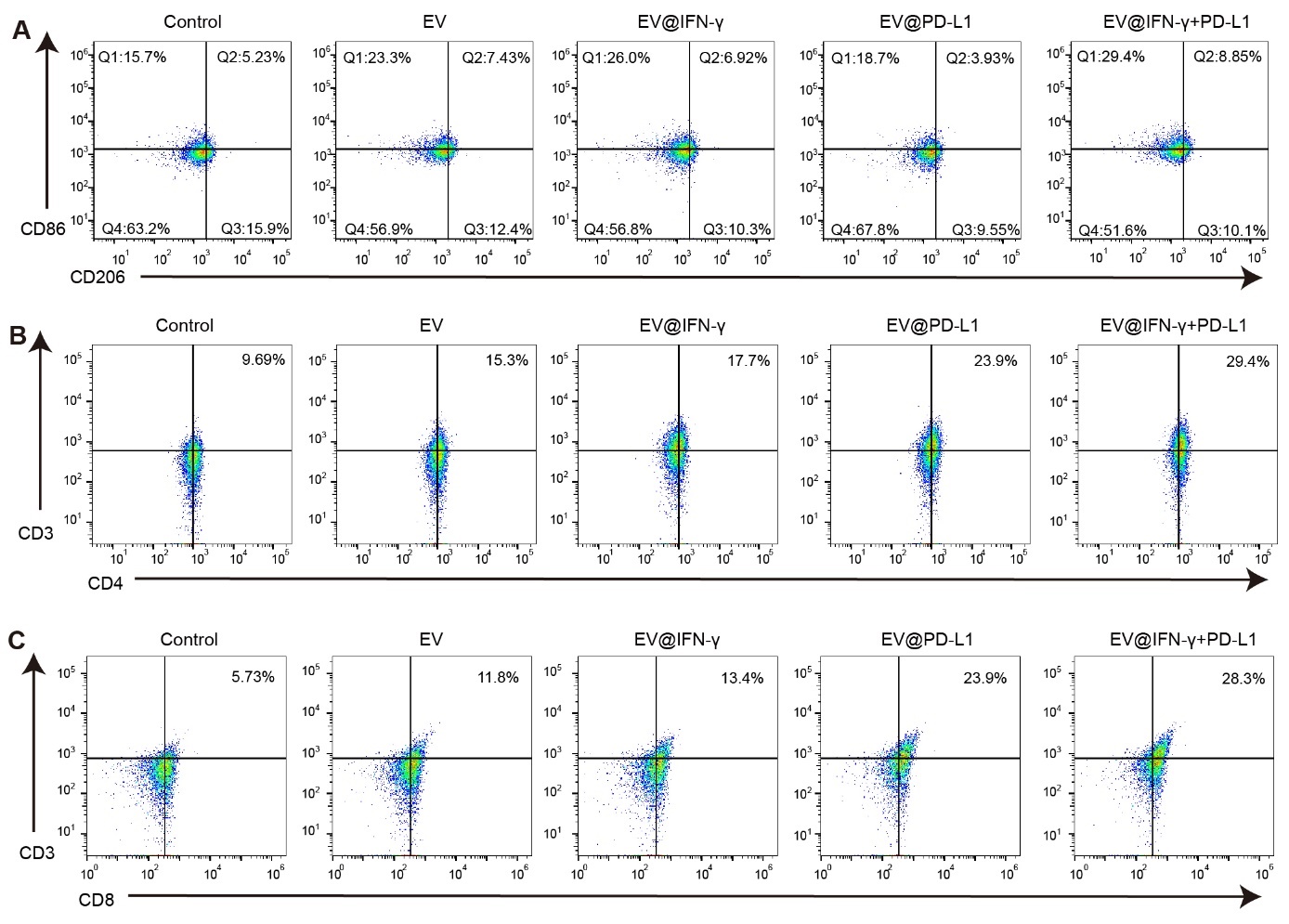


**Figure** **S21.** Characterization of macrophage phenotypes and activation of T cells in blood after treatment with SEE-EVs in PDX model. A) FACS scatter plot analysis of the expression of CD86 (marker of M1 macrophages) and CD206 (marker of M2 macrophages). B, C) FACS scatter plot analysis of the infiltration of CD4^+^ T cells (B) and CD8^+^ T cells (C). The data were from three independent experiments and were presented as mean ± SD.


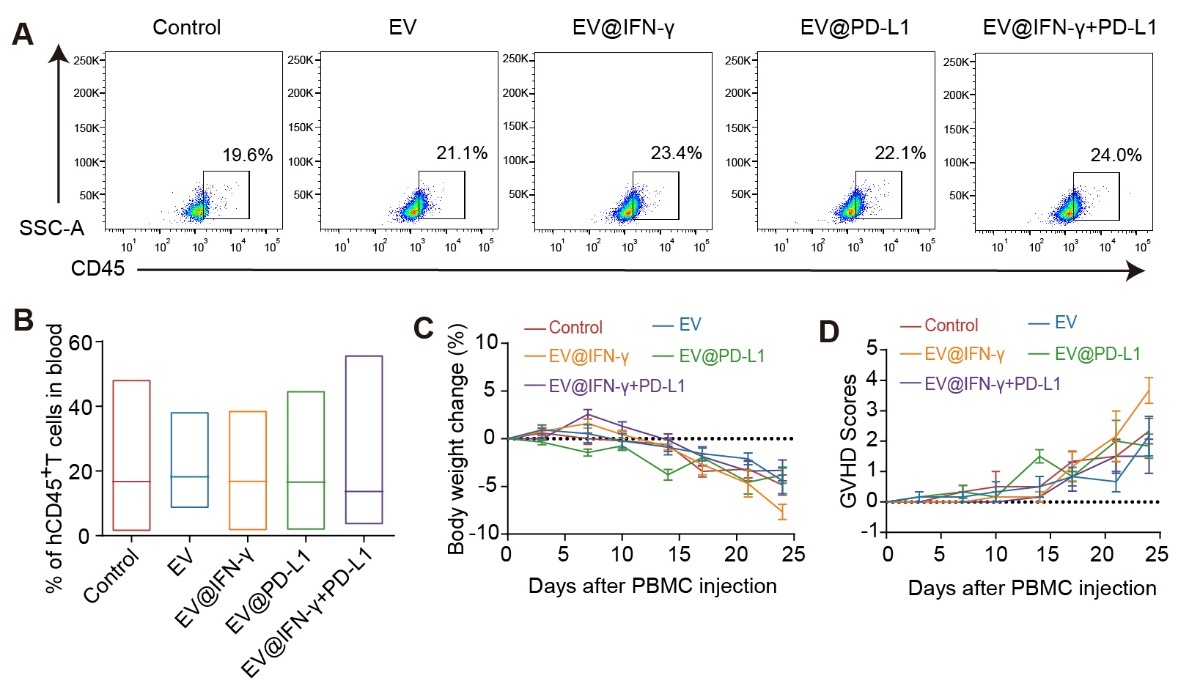


**Figure S22.** Reconstruction of human immune system. A) FACS analysis of the expression of CD45^+^ T cells in blood. B) Quantitative analysis of CD45^+^ T cells in blood for each mouse corresponding to (A). C) Weight change in humanized mice. D) Disease score in humanized mice. n = 6 mice per group.


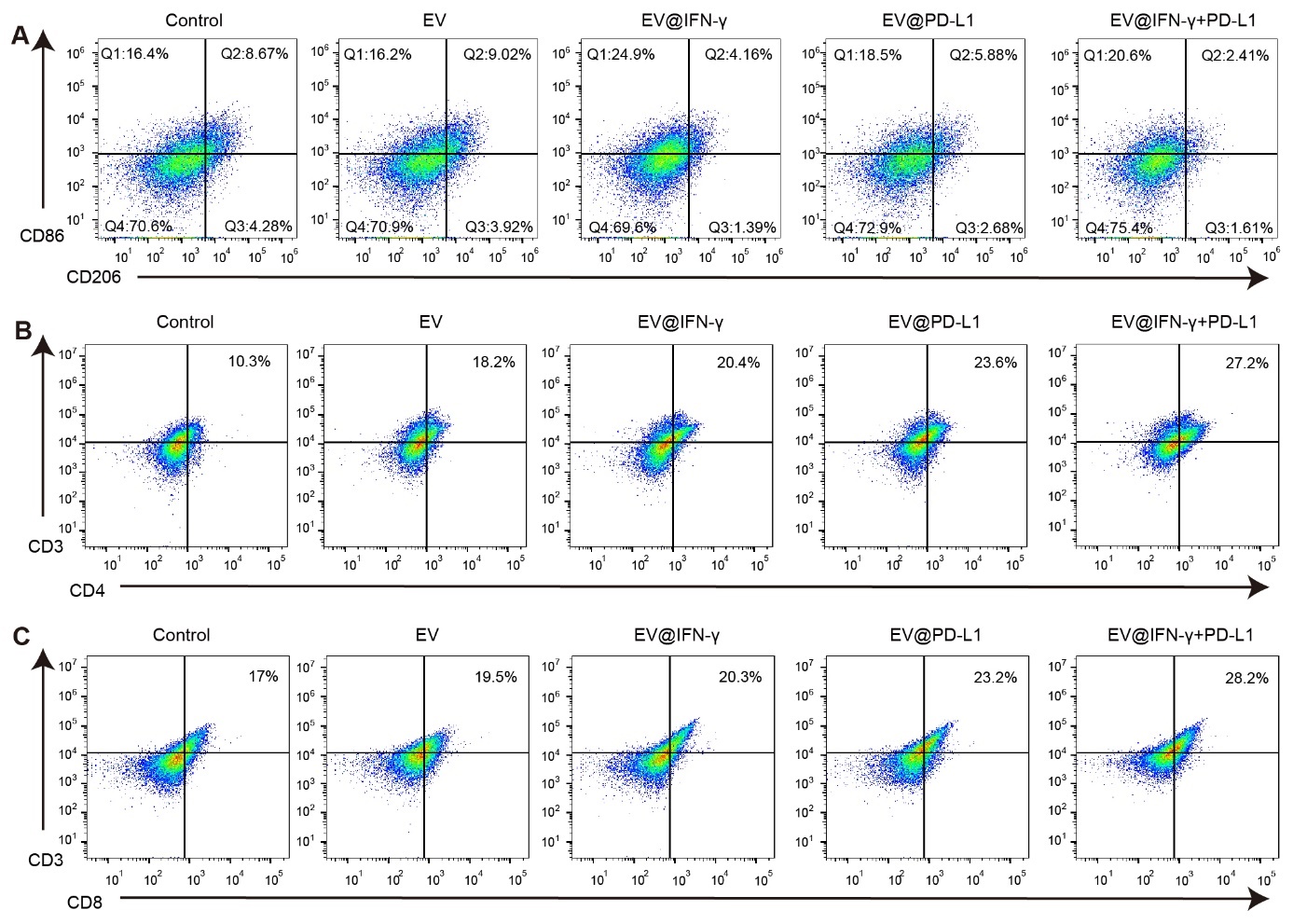


**Figure S23.** Characterization of macrophage phenotypes and activation of T cells in tumor tissues after treatment with SEE-EVs in PDX model. A) FACS scatter plot analysis of the expression of CD86 (marker of M1 macrophages) and CD206 (marker of M2 macrophages) in tumor tissues. B, C) FACS scatter plot analysis of the infiltration of CD4^+^ T cells (B) and CD8^+^ T cells (C). The data were from three independent experiments and were presented as mean ± SD.

**Supplementary Table 1. The sequence of primers, siRNA and plasmid.**

| Name | Sequence (5’-3’) |
| --- | --- |
| PDL1-Mouse-F | GCTCCAAAGGACTTGTACGTG |
| PDL1-Mouse-R | TGATCTGAAGGGCAGCATTTC |
| PDL1-Human-F | TGGCATTTGCTGAACGCATTT |
| PDL1-Human-R | TGGCATTTGCTGAACGCATTT |
| IFN-γ-Mouse-F | ATGAACGCTACACACTGCATC |
| IFN-γ-Mouse-R | CCATCCTTTTGCCAGTTCCTC |
| IFN-γ-Human-F | CTAGCGCCACCATGAAATATACAAGTT |
| IFN-γ-Human-R | CCGGTGGCTGGGATGCTCTTCGACCTC |
| U6-Mouse-F | GCTTCGGCAGCACATATACTAAAAT |
| U6-Mouse-R | CGCTTCACGAATTTGCGTGTCAT |
| U6-Human-F | ATTGGAACGATACAGAGAAGAT |
| U6-Human-R | GGAACGCTTCACGAATTT |
| GAPDH-Mouse-F | AGGTCGGTGTGAACGGATTTG |
| GAPDH-Mouse-R | TGTAGACCATGTAGTTGAGGTCA |
| GAPDH-Human-F | GTCTCCTCTGACTTCAACAGCG |
| GAPDH-Human-R | ACCACCCTGTTGCTGTAGCCAA |
| siPDL1-Mouse-Sense | ACUCAAAAUAAAUAGAAGGCA |
| siPDL1-Mouse-Antisense | CCUUCUAUUUAUUUUGAGUCU |
| siPDL1-Human-Sense | AAAUUGUAACAUCUACUACAA |
| siPDL1-Human-Antisense | GUAGUAGAUGUUACAAUUUUG |
| IFN-γ plasmid | Atgaaatatacaagttatatcttggcttttcagctctgcatcgttttgggttctcttggctgttactgccaggacccatatgtaaaagaagcagaaaaccttaagaaatattttaatgcaggtcattcagatgtagcggataatggaactcttttcttaggcattttgaagaattggaaagaggagagtgacagaaaaataatgcagagccaaattgtctccttttacttcaaactttttaaaaactttaaagatgaccagagcatccaaaagagtgtggagaccatcaaggaagacatgaatgtcaagtttttcaatagcaacaaaaagaaacgagatgacttcgaaaagctgactaattattcggtaactgacttgaatgtccaacgcaaagcaatacatgaactcatccaagtgatggctgaactgtcgccagcagctaaaacagggaagcgaaaaaggagtcagatgctgtttcgaggtcgaagagcatcccagtaa |

**Supplementary Table 2. The information of antibodies used in western blot.**

| Antibody name | Application | Catalogue | Manufacturer |
| --- | --- | --- | --- |
| Beta Actin rabbit polyclonal antibody | 1:5000 | AF7018 | Affinity Biosciences |
| CD63 rabbit polyclonal antibody | 1:1000 | AF5117 | Affinity Biosciences |
| Interferon gamma rabbit polyclonal antibody | 1:1000 | AF5183 | Affinity Biosciences |
| PD-L1 mouse monoclonal antibody | 1:1000 | BF8035 | Affinity Biosciences |
| CD9 rabbit monoclonal antibody | 1:1000 | ET1601-9 | Huaan Biotechnology |
| TSG101 rabbit monoclonal antibody | 1:2000 | ET1701-59 | Huaan Biotechnology |
| HRP-Goat anti-mouse secondary | 1:2000 | 7076 | Cell Signaling Technology |
| HRP-Goat anti-rabbit secondary | 1:2000 | 7074 | Cell Signaling Technology |

**Supplementary Table 3****. The information of clinical patients that provided tumor tissue samples for PDX model.**

| Number | Sex | Age | Tumor type | TNM Phase | Total phase | Application |
| --- | --- | --- | --- | --- | --- | --- |
| 1 | Women | 77 | Adenocarcinoma of the lungs | pT2bN0M0 | IIA | tumor metastatic inhibition |
| 2 | Women | 60 | Adenocarcinoma  of the lungs | pT1cN0M0 | IA | tumor metastatic inhibition |
| 3 | Men | 40 | Adenocarcinoma of the lungs | pT1bN0M0 | IA | tumor metastatic inhibition |
| 4 | Men | 72 | Adenocarcinoma of the lungs | pT1cN2M0 | IIIA | tumor metastatic inhibition |
| 5 | Men | 58 | Adenocarcinoma of the lungs | pT1bN0M0 | IA | tumor metastatic inhibition |
| 6 | Women | 77 | Adenocarcinoma of the lungs | pT1miN0 | IA | tumor metastatic treatment |
| 7 | Men | 65 | Adenocarcinoma of the lungs | pT2N2M0 | IIIA | tumor metastatic treatment |
| 8 | Men | 46 | Adenocarcinoma of the lungs | pT2aN2M0 | IIIA | tumor metastatic treatment |
| 9 | Men | 69 | Adenocarcinoma of the lungs | pT2aN2M0 | IIIA | tumor metastatic treatment |
| 10 | Men | 52 | Adenocarcinoma of the lungs | pT2aN2M0 | IIIA | tumor metastatic treatment |

**Supplementary Table** **4. The information of antibodies used in flow cytometry.**

| Antibody name | Application | Catalogue | Manufacturer |
| --- | --- | --- | --- |
| Anti-Mouse CD16/32 Antibody | 1:20 | E-AB-F0997A | Elabscience |
| Anti-Human CD16 Antibody | 1:20 | E-AB-F1236A | Elabscience |
| Elab Fluor® Violet 450 Anti-Mouse CD45 Antibody | 1:20 | E-AB-F1136Q | Elabscience |
| PE/Cyanine7 Anti-Mouse CD3 Antibody | 1:20 | E-AB-F1013H | Elabscience |
| FITC Anti-Mouse CD4 Antibody | 1:20 | E-AB-F1097C | Elabscience |
| PerCP/Cyanine5.5 Anti-Mouse CD8a Antibody | 1:20 | E-AB-F1104J | Elabscience |
| Elab Fluor® Violet 450 Anti-Human CD45 Antibody | 1:20 | E-AB-F1137Q | Elabscience |
| PE/Cyanine7 Anti-Human CD3 Antibody | 1:20 | E-AB-F1001H | Elabscience |
| FITC Anti-Human CD4 Antibody | 1:20 | E-AB-F1109C | Elabscience |
| PerCP/Cyanine5.5 Anti-Human CD8a Antibody | 1:20 | E-AB-F1110J | Elabscience |
| Elab Fluor®700 Anti-Mouse F4/80 Antibody | 1:20 | E-AB-F0995M1 | Elabscience |
| PE Anti-Mouse CD86 Antibody | 1:20 | E-AB-F0994D | Elabscience |
| APC Anti-Mouse CD206/MMR Antibody | 1:20 | E-AB-F1135E | Elabscience |

expanding the range of deliverable molecules to enhance therapeutic efficacy through multiple mechanisms.

**
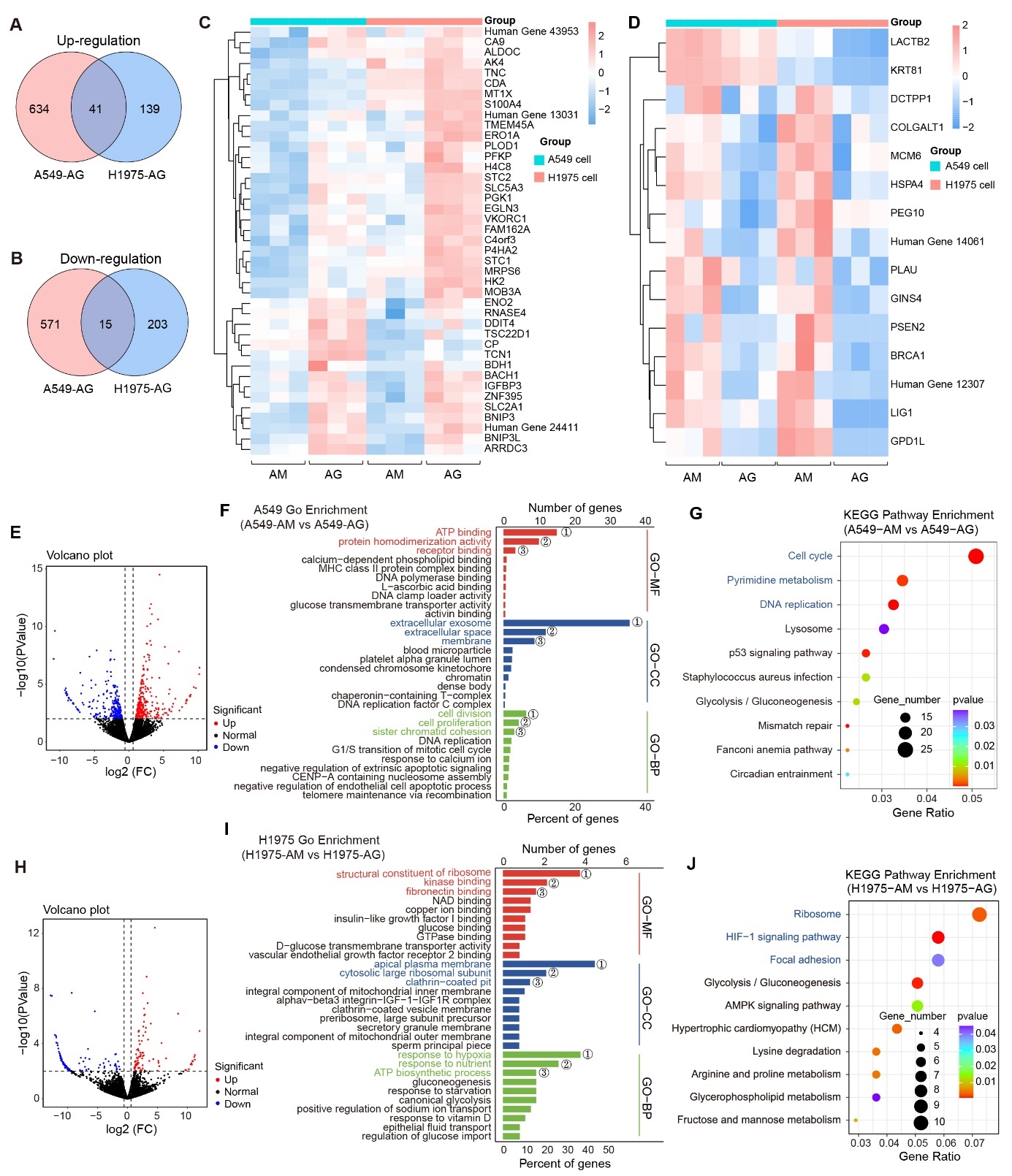
**

**Extended Data Figure 1.** Differential gene expression analysis of cancer cell subpopulations after evolutionary screening. A, B) Venn diagram of up-(A) and downregulated (B) genes in AG cells *vs.* AM cells. The networks represent the enriched terms (colored by cluster identification). The left circles represent the number of up-regulated (A) and down-regulated (B) genes in A549-AG *vs*. A549-AM, respectively. The right circles represent the number of up-regulated (A) and down-regulated (B) genes in H1975-AG *vs*. H1975-AM, respectively. Overlapping segments represent the number of genes that are jointly up-regulated (A) and down-regulated (B) in the two groups. C, D) The up-(C) and downregulated (D) genes in AG cells *vs.* AM cells. E) Volcano plot showing the gene expression differences between A549-AM and A549-AG cells. F, G) GO (F) and KEGG (G) pathway enrichment analysis of differentially expressed genes between A549-AM and A549-AG cells. H) Volcano plot displaying the gene expression differences between H1975-AM and H1975-AG cells. I, J) GO (I) and KEGG (J) pathway enrichment analysis of differentially expressed genes between H1975-AM and H1975-AG cells. The screening criterion was fold-change > 1.5-fold and *p* < 0.05 were considered statistically significant. *P*-values were calculated using fisher’s exact test with the hypergeometric algorithm and adjusted using the Benjamini-Hochberg method for multiple tests (E-J).

**
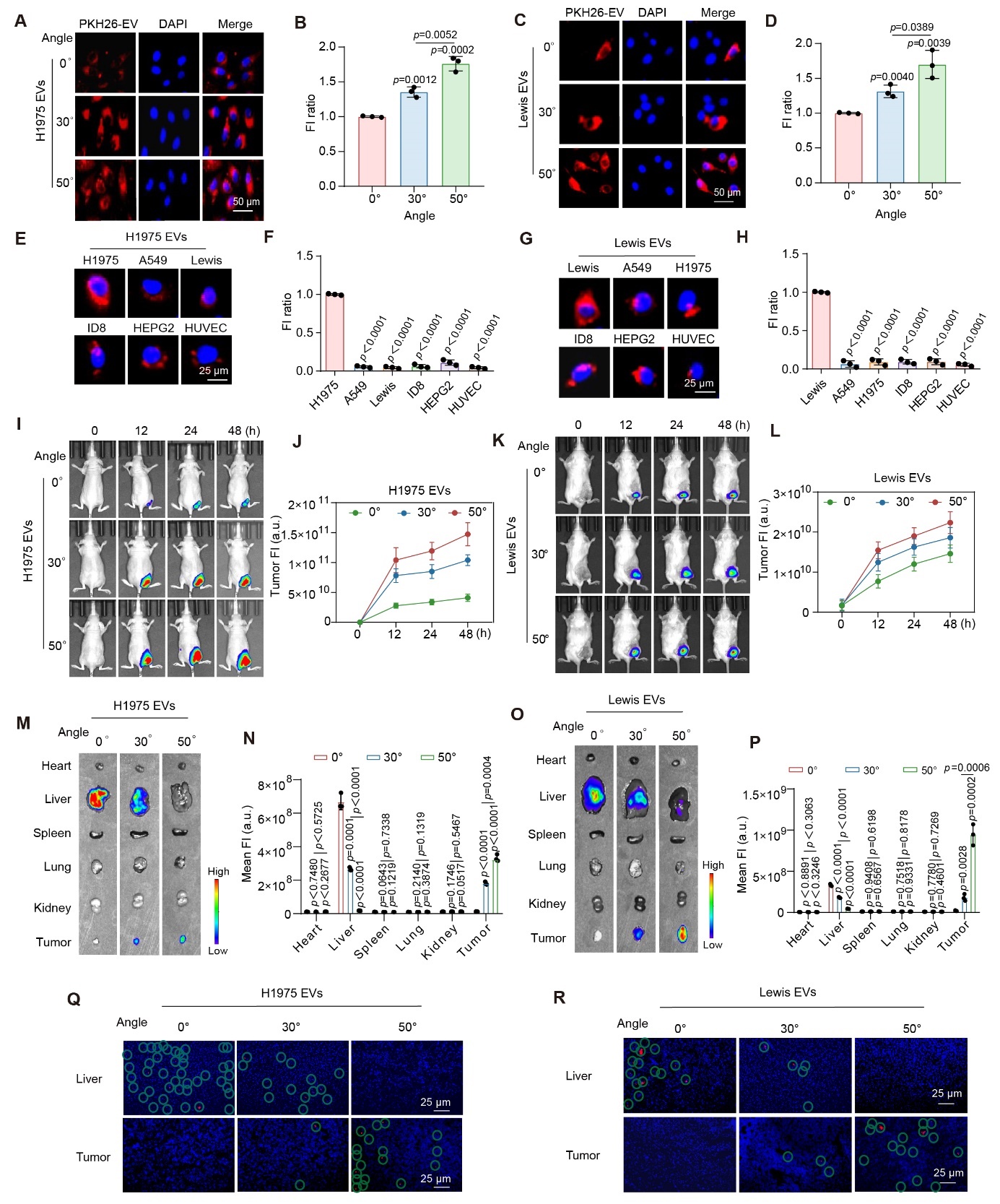
**

**Extended Data Figure 2.** Evolutionary screening enhanced the targeting of EVs to parental cells. A, B) Fluorescence microscopy images (A) and quantitative analysis (B) of the uptake of EVs by parental H1975 cells (scale bars = 50 μm). Red: PKH26-labeled EVs; Blue: DAPI-stained cell nuclei. C, D) Fluorescence microscopy images (C) and quantitative analysis (D) of the uptake of EVs by parental Lewis cells (scale bars = 50 μm). Red: PKH26-labeled EVs; Blue: DAPI-stained cell nuclei. E, F) Fluorescence microscopy images (E) and quantitative analysis (F) of the uptake of EVs-50° derived from H1975 cells by different cell types (scale bars = 25 μm). Red: PKH26-labeled EVs; Blue: DAPI-stained cell nuclei. G, H) Fluorescence microscopy images (G) and quantitative analysis (H) of the uptake of EVs-50° derived from Lewis cells by different cell types (scale bars = 25 μm). Red: PKH26-labeled EVs; Blue: DAPI-stained cell nuclei. I, J) *In vivo* distribution (I) and quantitative analysis (J) of EVs in H1975 tumor-bearing BALB/c nude mice at different time points post intravenous injection. K, L) *In vivo* distribution (K) and quantitative analysis (L) of EVs in Lewis tumor-bearing BALB/c mice at different time points post intravenous injection. M, N) *Ex vivo* fluorescence images (M) and quantitative analysis (N) of the main organs from H1975 tumor-bearing BALB/c nude mice. O, P) *Ex vivo* fluorescence images (O) and quantitative analysis (P) of the main organs from Lewis tumor-bearing BALB/c mice. Q, R) Frozen sections of the liver and tumor. Cell nuclei and EVs are shown in blue and red fluorescence (marked with green circles), respectively (scale bars = 25 μm). The data were derived from three independent experiments and were presented as mean ± SD; two-sided Student’s *t*-test was used for comparisons (B, D, F, H, N, P). *p* < 0.05 was considered statistically significant.


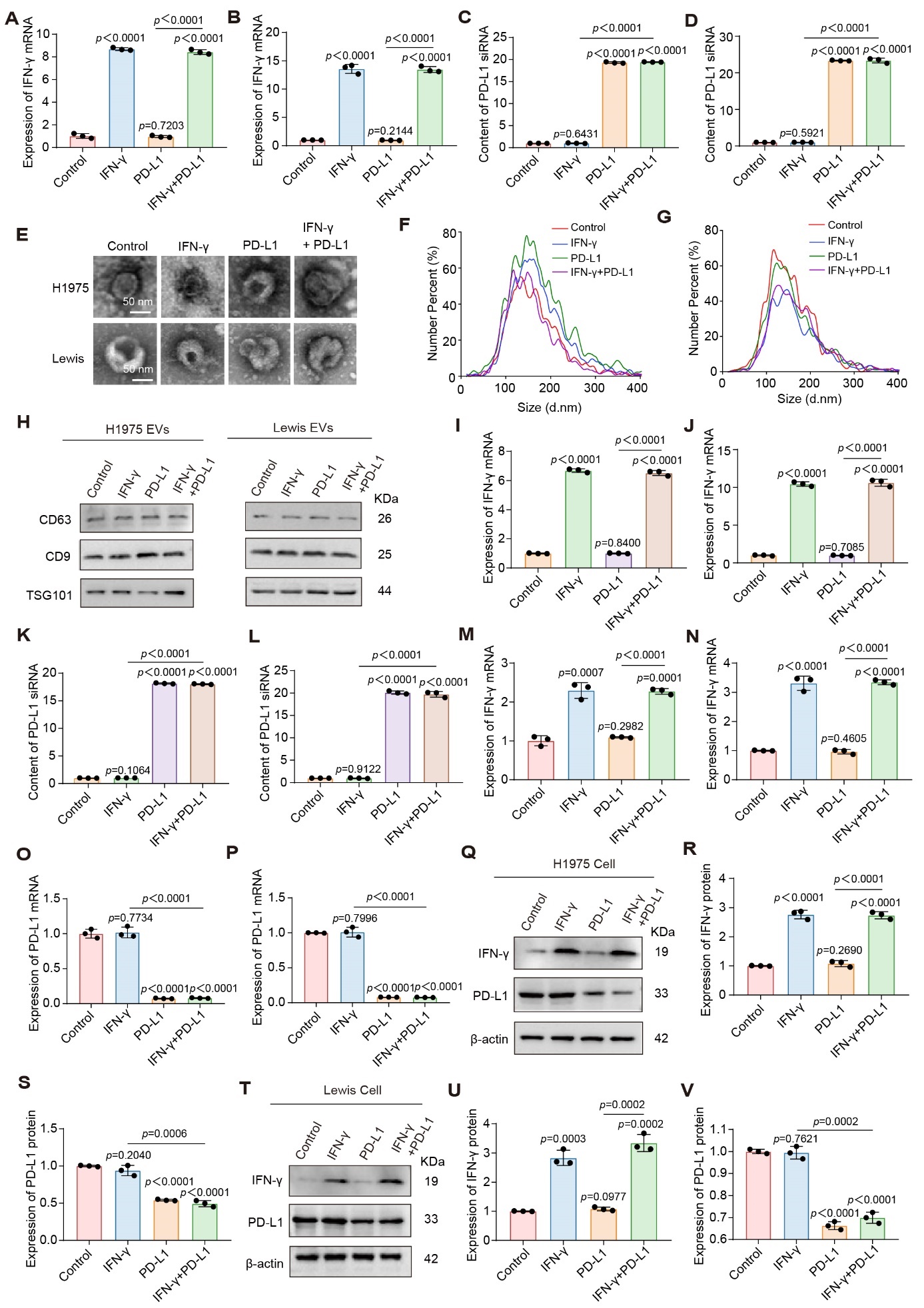


**Extended Data Figure 3.** Characterization of EVs endowed with therapeutic properties. A, B) qPCR analysis of IFN-γ mRNA in H1975 (A) and Lewis cells (B). C, D) qPCR analysis of PD-L1 siRNA in H1975 (C) and Lewis cells (D). Control: untreated cells; IFN-γ: cells loaded with the IFN-γ plasmid; PD-L1: cells loaded with PD-L1 siRNA; IFN-γ+PD-L1: cells loaded with both the IFN-γ plasmid and PD-L1 siRNA. E) TEM images of EVs derived from H1975 (top) and Lewis cells (bottom) (scale bars = 50 nm). (E-L) Control: EVs derived from untreated cells; IFN-γ: EVs derived from cells loaded with the IFN-γ plasmid; PD-L1: EVs derived from cells loaded with PD-L1 siRNA; IFN-γ+PD-L1: EVs derived from cells loaded with both the IFN-γ plasmid and PD-L1 siRNA. F, G) NTA analysis of EVs derived from H1975 (F) and Lewis cells (G). H) Western blot analysis of surface markers (CD63, CD9, and TSG101) on EVs derived from H1975 (left) and Lewis cells (right). I, J) qPCR analysis of IFN-γ mRNA in EVs derived from H1975 (I) and Lewis cells (J). K, L) qPCR analysis of PD-L1 siRNA in EVs derived from H1975 (K) and Lewis cells (L). M, N) qPCR analysis of IFN-γ mRNA in parental H1975 (M) and Lewis cells (N) after incubation with EVs. (M-V) Control: parental cells after incubation with EVs derived from untreated cells; IFN-γ: parental cells after incubation with EVs derived from cells loaded with the IFN-γ plasmid; PD-L1: parental cells after incubation with EVs derived from cells loaded with PD-L1 siRNA; IFN-γ+PD-L1: parental cells after incubation with EVs derived from cells loaded with both the IFN-γ plasmid and PD-L1 siRNA. O, P) qPCR analysis of PD-L1 mRNA in parental H1975 (O) and Lewis cells (P) after incubation with EVs. Q) Western blot analysis of IFN-γ and PD-L1 protein levels in parental H1975 cells after incubation with EVs. R, S) Quantitative analysis of IFN-γ (R) and PD-L1 (S) protein levels in parental H1975 cells after incubation with EVs. T) Western blot analysis of IFN-γ and PD-L1 protein levels in parental Lewis cells after incubation with EVs. U, V) Quantitative analysis of IFN-γ (U) and PD-L1 (V) protein levels in parental Lewis cells after incubation with EVs. The data were derived from three independent experiments and were presented as mean ± SD; two-sided Student’s *t*-test was used for comparisons (A-D, I-P, R, S, U and V). *p* < 0.05 was considered statistically significant.


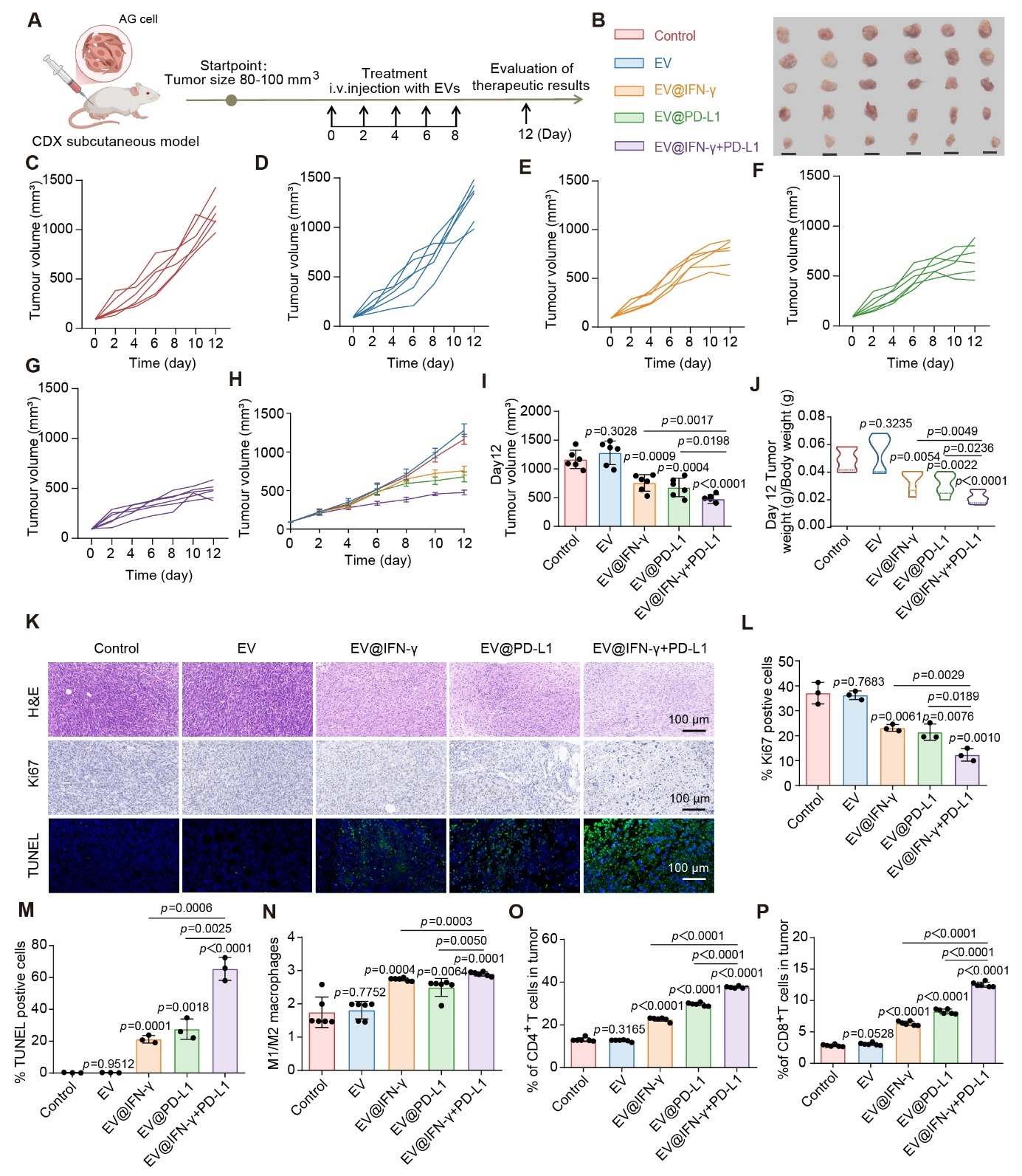


**Extended Data Figure 4.** Treatment of tumor metastasis using SEE-EVs in CDX model. A) Construction and therapeutic regimen of CDX model mice with metastatic tumors (n = 6 mice per group). B) Images showing the tumor size at the end of treatment. C-G) Tumor volume growth curves of the CDX model mice during the treatment process. Control (C), EV (D), EV@IFN-γ (E), EV@PD-L1 (F), and EV@IFN-γ+PD-L1 (G). H) Tumor volume growth curves of Lewis tumor-bearing mice in different treatment groups. I) Statistical analysis of the tumor volumes in each group at the end of treatment. J) Tumor to body weight ratios of different treatment groups. K) H&E (top), Ki67 (middle), and TUNEL staining (bottom) of tumors following different treatments (scale bars = 100 μm). L, M) Percentage of Ki67 (L) and TUNEL (M) positive cells among all cells at the end of treatment in each group. N) Phenotypic polarization of macrophages within tumors in different treatment groups. O, P) Percentage of CD4^+^ T cells (O) and CD8^+^ T cells (P) in different treatment groups. The data were derived from three independent experiments unless otherwise stated and were presented as mean ± SD; two-sided Student’s *t*-test was used for comparisons (I, J and L-P). *p* < 0.05 was considered statistically significant.


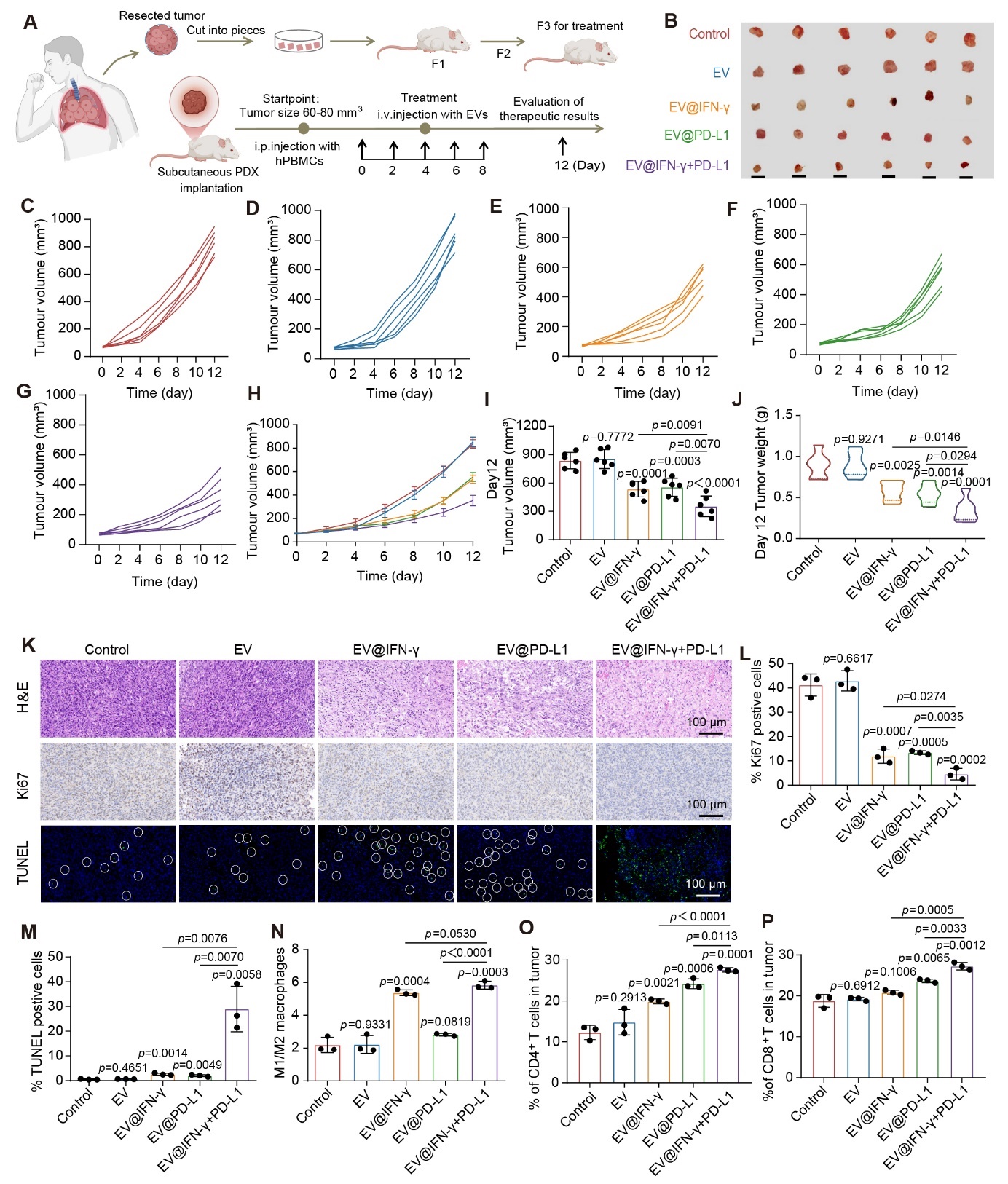


**Extended Data Figure 5**. Treatment of tumor metastasis using SEE-EVs in PDX model. A) Construction and therapeutic regimen of PDX model mice with metastatic tumors (n = 6 mice per group). B) Images showing the tumor size at the end of treatment. C-G) Tumor volume growth curves of PDX model mice during the treatment process. Control (C), EV (D), EV@IFN-γ (E), EV@PD-L1 (F), and EV@IFN-γ+PD-L1 (G). H) Tumor volume growth curves of PDX model mice in different treatment groups. I) Statistical analysis of tumor volumes in each group at the end of treatment. J) The weight of tumors in different treatment groups. K) H&E (top), Ki67 (middle), and TUNEL staining (bottom) of tumors following different treatments (scale bars = 100 μm). The white circles indicate the location of TUNEL-positive cells. L, M) Percentage of Ki67 (L) and TUNEL (M) positive cells among all cells at the end of treatment in each group. N) Phenotypic polarization of peripheral blood macrophages in different treatment groups. O, P) Percentage of CD4^+^ T cells (O) and CD8^+^ T cells (P) in different treatment groups. The data were derived from three independent experiments unless otherwise stated and were presented as mean ± SD; two-sided Student’s *t*-test was used for comparisons (I, J and L-P). *p* < 0.05 was considered statistically significant.
